# Absolute Binding Free Energy Calculations between the SARS-CoV‑2 Main Protease and 130 Drug Leads Using Implicit Ligand Theory

**DOI:** 10.1021/acs.jcim.6c00077

**Published:** 2026-05-20

**Authors:** Hong Ha Nguyen, Bing Xie, David D. L. Minh

**Affiliations:** Department of Chemistry, 2455 Illinois Institute of Technology, Chicago, Illinois 60616, United States

## Abstract

Absolute binding free energy (Δ*G*) calculations can rank structurally diverse compounds, which could be useful for early-stage drug discovery. Unfortunately, for flexible systems, it can be challenging to sample the receptor conformations necessary to obtain converged Δ*G* calculations. Here, we address this challenge by leveraging extensive molecular dynamics simulations of apo SARS-CoV-2 main protease (MPro) that were conducted on the Folding@Home distributed computing system. A Markov state model (MSM) was built to compute the equilibrium probability of each snapshot. Representative snapshots were selected from clusters defined based on occupancy fingerprints of the catalytic site. The binding potential of mean force (BPMF), the binding free energy between a ligand and *rigid* receptor configuration, was computed between the representative snapshots and 130 drug leads from the COVID Moonshot, an open-source drug discovery project. Δ*G*s were computed using an exponential average of BPMFs based on implicit ligand theory (ILT). Δ*G* calculations recapitulated experimental values with a Pearson R of 0.55 and a mean-adjusted root-mean-square error of 1.6 kcal/mol. Accuracy and computational costs were found to be intermediate between docking and previous free energy calculations with a fully flexible receptor. Moreover, in 88% of systems, the calculated Δ*G* of the native binding pose (RMSD from crystallographic <3 Å) was within 1 kT of the top-ranked pose.

## Graphical abstract

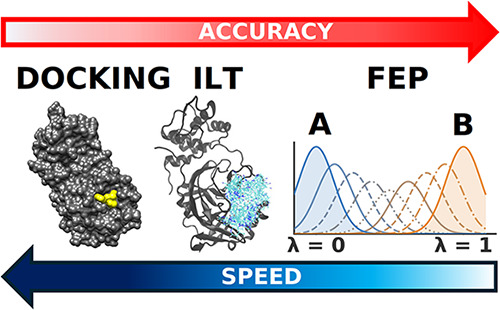

## Introduction

Drug discovery is a resource-intensive process that could be made more efficient by using fast and accurate computational methods for calculating binding free energies. A critical early step in most discovery programs is the identification of hit compounds, molecules that exhibit some level of biological activity. Hit identification can be achieved through high-throughput screening of large chemical libraries. However, high-throughput screening is expensive and often yields many false positives,^1^ including compounds that interfere with the assay through fluorescence or redox artifacts,^2^ bind promiscuously to multiple targets, form colloidal aggregates,^3,4^ or are pan-assay interference compounds.^5,6^ High-throughput screening may be supplemented or even replaced by virtual screening, its *in silico* counterpart.^7–9^ The cornerstone of virtual screening methods for hit discovery is molecular docking,^10^ which uses protein–ligand interaction energies to rapidly predict binding poses and free energies. While docking can be helpful for prioritizing compounds for synthesis and testing, it does not accurately forecast binding free energies.^11^ Secondary virtual screening with more accurate binding free energy calculations could increase the hit rate – the fraction of tested compounds that demonstrate biological activity – and increase the quality of hits.

Accurate free energy calculations can be achieved through free energy perturbation (FEP) methods^12^ that incorporate more rigorous treatments of entropy and solvation than docking. Relative binding free energy (ΔΔ*G*) calculations based on FEP are routinely used to design analogues during the hit-to-lead and lead optimization stages of drug discovery,^13,14^ and have demonstrated excellent accuracy in predicting relative binding affinities within congeneric series.^15–17^ For the identification of hits from diverse libraries, absolute binding free energy (Δ*G*) calculations that do not require a reference compound are more suitable. However, accurate absolute binding free energy (Δ*G*) calculations can be considerably more challenging; more extensive simulation is required to sample differences between unbound and bound thermodynamic states.^18^ Unfortunately, the computational expense of Δ*G* calculations via FEP is prohibitive for large-scale or even secondary virtual screening.^19^

Implicit ligand theory (ILT) is the basis of a possible strategy to bridge the gap between fast but inaccurate docking methods and accurate but slow FEP methods for Δ*G*. According to ILT, which our senior author initially published in 2012,^20^ Δ*G* is an exponential average of the binding potential of mean force (BPMF), the absolute binding free energy between a flexible ligand and rigid receptor configuration, over the apo ensemble of receptor configurations. Later, our group derived variants of ILT to compute ΔΔ*G* based on exponential averages of BPMFs over a holo ensemble.^21,22^ A software package that represents receptor–ligand interactions on a precomputed alchemical grid, AlGDock,^23^ was developed to efficiently calculate BPMFs. Instead of repetitively computing pairwise interactions, AlGDock interpolates these grids, significantly accelerating energy evaluations compared to methods that use fully flexible receptors.

The performance of ILT-based calculations is highly dependent on the selection of receptor configurations. By applying AlGDock to an ensemble of receptor snapshots extracted from fully flexible FEP calculations for a small subset of ligands, Xie et al.^24^ were able to accurately and efficiently calculate Δ*G* between T4 lysozyme L99A, in which the mutation produces a small hydrophobic cavity in a fairly rigid protein, and 141 fragment-like organic molecules. In contrast, in the Drug Design Data Resource Grand Challenge 3 (Subchallenge 2),^25^ the performance of AlGDock was system-dependent. Due to an indexing error in the procedure for selecting receptor configurations, the calculations submitted to the blinded prediction challenge were based on a random subset of experimentally determined receptor structures from the Protein Data Bank. With the vascular endothelial growth factor receptor 2, where the subset was fortuitously diverse, AlGDock calculated one of the highest correlations with experimental free energies among submissions to the subchallenge. In other systems where the subset was less representative, the performance of AlGDock was relatively poor.

Our team has identified receptor configuration selection strategies with a high efficiency of stratification.^26^ Molecular dynamics simulations (MDS) and high-resolution structure determination techniques, including X-ray crystallography, cryogenic electron microscopy, and nuclear magnetic resonance, can generate many physically plausible configurations of a receptor. These configurations may be used in ensemble docking^27,28^ – performing molecular docking (or BPMF) calculations to multiple rigid configurations – as a strategy to account for receptor flexibility. However, performing calculations for all available receptor configurations can be computationally prohibitive. As ligands have similar binding affinities to similar receptor configurations, large-scale calculations can be made efficient by ensemble reduction, selecting a representative subset of configurations.^29^ While various ensemble reduction strategies have been developed, e.g., refs 29,30 it is not always clear which method should be selected.^31^ Recognizing that ensemble reduction can be framed as a statistical variance reduction technique known as stratified sampling, our team applied a quality metric, the efficiency of stratification, to various clustering algorithms for ensemble reduction.^26^ We showed that among these tested algorithms, those incorporating the Jaccard distance between occupancy fingerprints combined with hierarchical clustering were the most efficient, outperforming simple random sampling of the full receptor ensemble.

Here, we use occupancy fingerprint clustering to systematically capture binding site diversity for ILT-based Δ*G* calculations between a flexible receptor and ligands with a broad range of affinity. The receptor is the SARS-CoV-2 main protease (MPro), an essential enzyme in the viral life cycle that is targeted by the FDA-approved antiviral drug nirmatrelvir (Paxlovid).^32^ The ligands are 130 drug leads from the COVID Moonshot,^16^ an open-source drug discovery project. The COVID Moonshot has publicly released high-resolution structures of receptor–ligand complexes, biochemical inhibition data, and results from fully flexible FEP calculations for drug leads from throughout the campaign. For simplicity, we will refer to Δ*G* from the COVID Moonshot as FEP and our new calculations as AlGDock. We demonstrate that AlGDock can deliver accuracy close to FEP while remaining computationally tractable for secondary virtual screening. This work establishes a practical middle ground balancing speed and rigor, addressing a critical gap in early-stage drug discovery.

## Methods

For precision, let us define the way that we use common terms in this paper. A *configuration* describes a set of three-dimensional positions of atoms. A *structure* is a configuration modeled based on experimental data. A *snapshot* is a configuration sampled using molecular simulation methods. We will consider configurations of the receptor, ligand, and complex. A *conformation* is a set of similar configurations defined based on a clustering method. We will consider conformations of a complete *receptor*, receptor *binding site*, and receptor–ligand complex (a *binding pose*). A *native pose* has an RMSD < 3 Å from a (crystallographic) structure. We refer to ligand poses associated with a specific receptor configuration as *subposes*.

A brief summary of our methods is as follows: starting with Folding@Home MDS of apo MPro,^33^ we used a Markov state model (MSM) to assign equilibrium probabilities to each receptor conformation, which are evenly divided among snapshots within the conformation; representative snapshots were selected from binding site clusters based on occupancy fingerprints; ligands were docked to the representative receptor snapshots; BPMFs were calculated for a subset of representatives using three snapshot selection schemes; and Δ*G* were calculated as a weighted exponential average of BPMFs (Figure 1).

**Figure 1:**
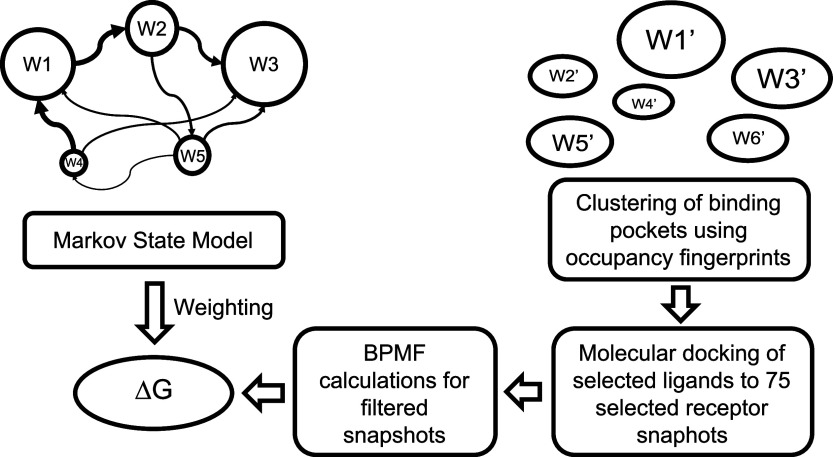
Schematic representation of the workflow for Δ*G* calculations using AlGDock in this report.

### Receptor Snapshot Selection and Weighting

Our free energy calculations were based on snapshots of apo MPro extracted from extensive simulations (2.9 ms) previously performed on the Folding@home distributed computing system.^34^ We used two independent clustering methods: one to estimate equilibrium probabilities of every snapshot and another to define binding site conformations and select representative snapshots.

Equilibrium probabilities were estimated on the basis of an MSM of apo MPro. The MSM was built similarly to our previous paper^35^ based on 20,798,069 snapshots from simulations performed on the Folding@home distributed computing platform.^33^ The MSM construction process differed from using a density-based clustering method to allow for irregularly shaped microstates. Specifically, snapshots were clustered into 91 microstates by applying the density-based method, adaptive partitioning by local density peaks (APLoD),^36^ to time-lagged independent components analysis. The lag time was selected based on implied time scale analysis; the lag time should be long enough for the system to forget its history and for calculated relaxation times to converge. The equilibrium probability of each snapshot (*w*_*s*_(*r*_*j*_) for receptor snapshot *r*_*j*_) was calculated by evenly distributing the equilibrium probability of each microstate (*w*_*m*_(*k*) for microstate *k*), as shown in eq 1

1 $$w_{s}\left(r_{j}\right)=\frac{w_{m}\left(m\left(r_{j}\right)\right)}{n\left(m\left(r_{j}\right)\right)}$$

where *m*(*r*_*j*_) is the microstate that snapshot *r*_*j*_ belongs to and *n*(*m*) is the number of snapshots in microstate *m*.

Representatives of binding pocket conformations were randomly selected from 75 clusters based on occupancy fingerprints^26^ of the binding site described our previous work.^35^ We previously showed that, according to principal components analysis of occupancy fingerprints, receptor conformations from the Folding@home simulations are a superset of those accessed in 806 crystallographic complexes.^35^ Thus, we anticipated that representatives of binding pocket conformations would be capable of binding all of our ligands. Weights of the representative snapshots of each binding site, *w*_*b*_(*r*_*j*_), were based on the sum of equilibrium probabilities of all snapshots within the cluster (eq 2)­

2 $$w_{b}\left(r_{j}\right)=\sum_{r_{j}\in b}w_{s}\left(r_{j}\right)$$

where the sum is over all snapshots with the binding site in conformation *b*. The weights were normalized such that ∑_*b*_w_*b*_ = 1.

### Ligand Selection

Over two rounds of calculations, we prepared a total of 130 ligands. Initially, 57 small molecules were randomly selected from the 631 that were cocrystallized with the enzyme as part of the COVID Moonshot.^16^ However, we later recognized that Boby et al.^16^ only performed FEP calculations for 37 of these selected molecules. To enable a more statistically significant comparison between the performance of our methods and the reported FEP calculations, we randomly selected an additional 73 molecules for which Boby et al.^16^ both solved crystal structures and performed FEP calculations. For each ligand, SMILES^37^ strings and crystallographic structures of MPro complexes were downloaded from Fragalysis (https://fragalysis.diamond.ac.uk/viewer/react/preview/target/Mpro). The chemical structures, FEP results, and experimental binding free energies of each ligand are described in Table S1 of the Supporting Information. Experimental binding free energies were converted from IC_50_ of enzyme activity assays based on the competitive inhibition assumption, Δ*G* = −*RT* ln *IC*_50_, in which *R* = 1.987 × 10^–3^ kcal·mol^–1^K^–1^ and *T* = 300 K.

### Molecular Docking

The 130 ligands were docked to the 75 receptor snapshots. Using MDAnalysis v2.3.0,^38,39^ the 75 snapshots were aligned to PDB ID 6Y2E to minimize the root-mean-square deviation between heavy atoms of residues in the binding pocket: residues 38 to 42, 110 to 148, and 162 to 166. The 130 crystallographic protein–ligand complexes were aligned using the same procedure to define the binding site center, calculated as the center of mass of all aligned ligand poses. The aligned systems were translated by the vector (−40, −58, −10) such that the binding site center is at (15, 15, 15). Protonation states of the proteins were maintained as they were in the molecular dynamics simulations performed on Folding@home;^34^ the simulations used appropriate protonation states for residues essential for the activity of MPro, including at the ε nitrogen of Histidine 41 (in catalytic dyad), 163, and 172.^35,40–42^

Docking was performed using UCSF DOCK6 v6.6 ^43^ (DOCK6) and AutoDock Vina v1.2.6 (Vina).^44^ DOCK6 results were used both for Δ*G* and pose prediction, but Vina was only used for pose prediction. Binding site parameters, including a center at (15, 15, 15) and sphere radius of 15 Å, were shared among both docking methods. For DOCK6, ligand models were parametrized based on the AMBER GAFF2 force field^45^ following the protocol described in Xie, Nguyen, and Minh (2017).^24^ Other parameters for DOCK6 were as previously described.^24^ For Vina, the exhaustiveness was set to 20 and the maximum number of output poses to 50.

### Snapshot Selection

Xie et al.^24^ observed that filtering receptor snapshots based on a docking score threshold achieves faster convergence compared to random selection. We also wanted to evaluate the possible benefit of leveraging crystallographic binding poses. Thus, we considered three schemes to select receptor snapshots to perform BPMF calculations:

(a) Docking;
(b) Full crystal;
(c) Random crystal;

The Docking scheme does not use any holo crystallographic information, but rather selects receptor snapshots based only on the minimum docking score *E*_*l*_ *(r*_*j*_) of each ligand *l* to the receptor snapshot *r*_*j*_. Only receptor snapshots where the ligand has a DOCK6 score below a specified threshold δ above this minimum were retained, *E*_*l*_(*r*_*j*_) < *min*(*E*_*l*_(*r*_*j*_)) + δ. We tested values of δ ∈ {5,8,10,12,15} kcal/mol in the Docking scheme. The Full Crystal scheme uses crystallographic structures of the receptor complexed with *every* ligand. The selection process is like the Docking scheme, except that the DOCK6 energy of crystallographic binding poses (opposed to *de novo* docking poses) of each ligand was minimized in each receptor snapshot. After rigid-body docking of the crystallographic ligand pose into the snapshot, minimization was performed in DOCK6. The simplex minimizer was used with step sizes of 1.0 Å (translation), 0.1 rad (rotation), and 10° (torsion) for up to 5000 iterations, terminating when the score change was less than 0.1 kcal mol^–1^. Because the minimized energy of these poses was highly sensitive to positions of binding site atoms, we also tested δ of 20 and 30 kcal/mol with the Full Crystal Scheme. As crystallographic structures of the receptor complexed with every ligand are rarely available, we developed the Random Crystal scheme to evaluate the effect of using *some* crystallographic complexes. In this scheme, a random subset of ligands with *holo* crystallographic structures was chosen to guide the snapshot selection process. Receptor snapshots were selected using the same procedure as in the Full Crystal scheme, but based on these random subsets. The combined sets of snapshots selected based on the random ligands were used for free energy calculations with all ligands.

For the Random Crystal scheme, we used bootstrapping to evaluate the precision of free energy estimates as a function of the identity and number of selected ligands. Specifically, the standard deviation was calculated for free energy calculations based on 1000 sets of receptor snapshots, where each set comprised N ∈ {1,2,4,6,20} ligands randomly sampled with replacement from the 57 available ligands from the first round of ligand preparation.

### Free Energy Calculations

According to ILT, the absolute binding free energy for a ligand *i*, Δ*G*_*i*_, is an exponential average of BPMFs (B­(*r*_*R*_)) in the apo distribution.^20^ Independent BPMF calculations were performed between each ligand and selected receptor snapshots. The calculations were performed using AlGDock in a similar way as described.^23^ The binding site was defined as a sphere with a radius of 15 Å and centered at the average center of mass of all of the selected ligands: (15, 15, 15). In AlGDock, BPMF calculations may be initiated from a set of distinct binding poses. For the Docking and Random Crystal schemes, these initial poses were solely from DOCK6. For the Full Crystal scheme, initial poses also included the minimized crystal pose. In AlGDock, cycles specify the length of simulation, and BC (ligand tempering) and CD (interaction grid scaling) are labels for processes within the thermodynamic cycle.^23^ BPMF calculations were run for 15 cycles of BC and 20 cycles of CD. The thermodynamic state D was described by Full solvation.^23^

Absolute binding free energies based on ILT (Δ*G*_*i*_^*ILT*^) between MPro and each ligand were calculated by combining the weights of the binding site conformations, *w*_*b*_, with the BPMFs calculated for the representative snapshots,^20,23,24^ as shown in eq 3

3 $$\Delta G_{i}^{\mathrm{ILT}}=-k_{B}T\;\ln\left(\sum_{b}w_{b}e^{-B\left(r_{R}\right)/k_{B}T}\right)+k_{B}T\;\ln\left(\frac{VC^{o}}{8\pi^{2}}\right)$$

where *B*(*r*_*R*_) is the estimated BPMF for the representative receptor snapshot *r*_*R*_ from binding site *b*, *k*_*B*_ is Boltzmann’s constant, *T* = 300 K is the temperature, *C* ^o^ is the standard concentration of 1/1666 (Å^3^), and *V* is the volume of the binding site, defined as a sphere with a radius of 15 Å. For representative snapshots that were not selected, *B*(*r*_*R*_) was assumed to be infinite, such that they contribute no weight to the exponential average.

To enable a fair comparison with the COVID Moonshot,^16^ we also calculated Δ*G* based on ΔΔ*G* calculations to reference compounds. The COVID Moonshot^16^ performed ΔΔ*G* calculations, which may benefit from additional cancellation of error compared to Δ*G*. Among the 110 selected ligands for which ΔΔ*G* was calculated, three small molecules were reference ligands. The relative binding free energy (eq 4) based on ILT (ΔΔ*G*_*i*_^*ILT*^) between ligand *i* and its reference ligand (*ref*) was calculated as the difference between their calculated absolute binding free energies

4 $$\Delta \Delta G_{i,\mathrm{ref}}^{\mathrm{ILT}}=\Delta G_{i}^{\mathrm{ILT}}-\Delta G_{\mathrm{ref}}^{\mathrm{ILT}}$$

The COVID Moonshot^16^ reported absolute binding free energies obtained by adding estimated relative free energies to experimental absolute binding free energies of the reference compounds. Likewise, we calculated Δ*G*_*i*_^Δ*ILT*^ using eq 5, based on experimental binding free energies of reference ligands (Δ*G*_*ref*_^*exp*^)­

5 $$\Delta G_{i}^{\Delta \mathrm{ILT}}=\Delta \Delta G_{i,\mathrm{ref}}^{\mathrm{ILT}}+\Delta G_{\mathrm{ref}}^{\exp}$$

Δ*G*_*i*_^Δ*ILT*^ computed using eq 5 based on ΔΔ*G* to reference compounds (eq 4) will be referred to with the prefix “Converted.”

### Pose Predictions

AlGDock defines binding poses by clustering ligand snapshots from the bound thermodynamic state in complex with *one* receptor configuration.^23^ The Pose BPMF (*B*_*i*_^*p*^(*r*_*R*_)) for ligand *i* in ligand pose *p* is calculated based on the weight of each pose, *w*_*p*_, in the fully bound state, using the following eq (eq 6)­

6 $${B_{i}^{p}\left(r_{R}\right)=B}_{i}\left(r_{R}\right)-k_{B}T\;\ln\left(\frac{w_{p}}{\sum_{p}w_{p}}\right)$$

The weight of the pose is calculated by the sum of the weights for each ligand snapshot clustered into the binding pose.^23^ Each pose is represented by the lowest-energy snapshot within the cluster.

As our present work considers BPMF calculations for multiple receptor snapshots, we evaluated two methods for pose prediction:

a) Receptor-weighted Pose BPMF: A free energy penalty for the probability of the binding site conformation *(w*_*b*_) of the receptor snapshot associated with pose *p* is added to the pose BPMF (eq 7),
7 $$B_{i}^{b,p}=B_{i}^{p}\left(r_{R}\right)-k_{B}T\ln\left(w_{b}\right)$$
  As with the pose BPMF, the pose is represented by the lowest-energy snapshot within the cluster.
b) Pose Δ*G*: Each pose can encompass multiple *subposes* – poses defined based on specific receptor snapshots. As described below, the free energy combines the holo probability of each subpose and the conformation with the lowest Receptor-weighted Pose PBMF was selected as the representative.

In the Pose Δ*G* method, subposes are combined based on the similarity of the ligand snapshot without regard for differences in the receptor conformation. Representative ligand snapshots from all BPMF calculations are clustered using agglomerative hierarchical clustering with complete linkage in SciPy v1.12.0.^46^ The representative conformation is represented by the lowest Receptor-weighted Pose BPMF within each cluster. The distance matrix is the symmetry-corrected root-mean-square deviation (RMSD) of these ligand snapshots, calculated using spyrmsd v0.8.0.^47^ Clusters are flattened using a distance threshold of 3 Å. The Pose Δ*G* (eq 8) is calculated by a weighted exponential average of subpose BPMFs *B*_*i*_^*s*^ across subposes within the pose, s ∈ p

$$\Delta G_{i}^{p}=-k_{B}T\;\ln\left(\underset{s\in p}{\sum }w_{b}\left(s\right)e^{-B_{i}^{s}/k_{B}T}\right)+k_{B}T\;\ln\left(\frac{VC^{o}}{8\pi^{2}}\right)$$ 8

where *w*_*b*_(*s*) is the apo probability of the binding site conformation associated with the subpose. The second term is due to standard state corrections, where *V* is the binding site volume, *C* ^o^ = 1 M is the standard concentration, and 8π^2^ is an integral over rotational degrees of freedom.^20,48^

The binding pose predictions of 130 systems by AlGDock were then compared to DOCK6 and Vina. Two scoring schemes, the **Receptor-weighted Pose BPMF** and **Pose Δ*G***, were applied to rank binding pose predictions of AlGDock, DOCK6, and Vina using eqs 7 and 8. For DOCK6 and Vina, the docking scores were used in place of the BPMF values in these equations. Snapshot selection schemes were not applied to DOCK6 and Vina.

### Correlation and Binary Classification Metrics

The accuracy of free energy predictions was evaluated using the Pearson R,^49^ Spearman ρ,^50^ Kendall τ,^51^ root-mean-square error (RMSE), and adjusted RMSD (aRMSD) using SciPy v1.12.0.^46^ These metrics were applied to compare binding free energies predicted by DOCK6, AlGDock, and FEP against experimental data, with both the FEP predictions and experimental data sourced from the COVID Moonshot database. The Pearson R assesses linear relationships, while the Spearman ρ and Kendall τ evaluate rank ordering, with the Spearman ρ being less sensitive to outliers. The RMSE is calculated using eq 9:

9 $$\mathrm{RMSE}=\sqrt{\frac{1}{N}\sum_{n=1}^{N}{\left(x_{n}-y_{n}\right)}^{2}.}$$

The aRMSE, accounting for both individual deviations and systematic bias, is defined as eq 10

10 $$\mathrm{aRMSE}=\sqrt{\frac{1}{N}\sum_{n=1}^{N}{\left[x_{n}-y_{n}-\left(\mathrm{x̅}-\mathrm{y̅}\right)\right]}^{2}}$$

where the *x̅* and *y̅* are the means of variables *x* and *y*, respectively. The aRMSE is particularly useful for evaluating the accuracy of relative binding free energy predictions.

Receiver operating characteristic (ROC) curves were employed to evaluate the ability of each method to distinguish high-affinity from low-affinity binders. Ligands were categorized as *stronger binders* if their experimental Δ*G* was more negative than the mean experimental value (−7.78 kcal/mol) across all systems. To quantify performance, the area under the curve (AUC) and semilog AUC (logAUC) values were calculated using scikit-learn v1.5.1,^52^ providing a measure of both global discriminative accuracy and early enrichment success.

All BPMF calculations were performed as single-core jobs on the Anvil computing system at Purdue University. We used standard shared CPU nodes (opposed to GPU or large-memory nodes), which comprise dual 64-core AMD Epyc “Milan” processors clocked at 2.45 GHz and interconnected via 100 Gbps Mellanox HDR InfiniBand. Each job required approximately 5 GB of RAM and consumed 15–24 h of CPU compute time.

## Results

### Density-Based Clustering Yields Similar Kinetics as K-Means Clustering with Fewer Microstates

Using the density-based clustering method APLoD with 91 microstates, we achieved a variational approach for Markov processes-2 (VAMP-2) score^53^ comparable to K-means clustering with 2500 microstates. Implied time scales plateau after 2 to 3 ns. The Chapman–Kolmogorov test for an MSM based on a lag time of 3 ns shows close agreement between estimated and predicted transition probabilities. For additional details, see ***Section B.1 in the*** Supporting Information.

### AlGDock Δ*G* Calculations Were More Accurate than Molecular Docking but Less Accurate than FEP

For the complete set of 130 ligands, AlGDock with the Full Crystal scheme achieved an RMSE of 4.97 kcal/mol and mean-adjusted RMSE (aRMSE) of 1.62 kcal/mol (Figure 2a). The difference between the RMSE and aRMSE shows that much of the error is systematic, as opposed to random. The AlGDock calculations have moderate correlation with experiment (Pearson R of 0.55, Spearman ρ of 0.54, and Kendall τ of 0.37). In contrast, DOCK6 scores (Figure 2b) showed markedly larger deviations in binding free energy, with an RMSE of 35.25 kcal/mol. Even after correcting for systematic shifts, the error remained high, with an aRMSE of 5.56 kcal/mol. Correspondingly, correlation metrics were weaker (*R* = 0.25, ρ = 0.29, τ = 0.20). Intriguingly, Vina (Figure 2c) achieved a low RMSE (1.28 kcal/mol) and aRMSE (0.99 kcal/mol) and comparable ranking performance (e.g., τ = 0.39) to AlGDock. However, predicted binding free energies were in a severely compressed range such that the linear regression slope was only 0.22. While keeping predictions within a narrow range lowers the RMSE within this particular benchmark, it fails to capture the true range of binding free energies. Consequently, AlGDock, with a slope of 0.93, provides a much more physically realistic and sensitive prediction of Δ*G* than either tested docking method.

**Figure 2:**
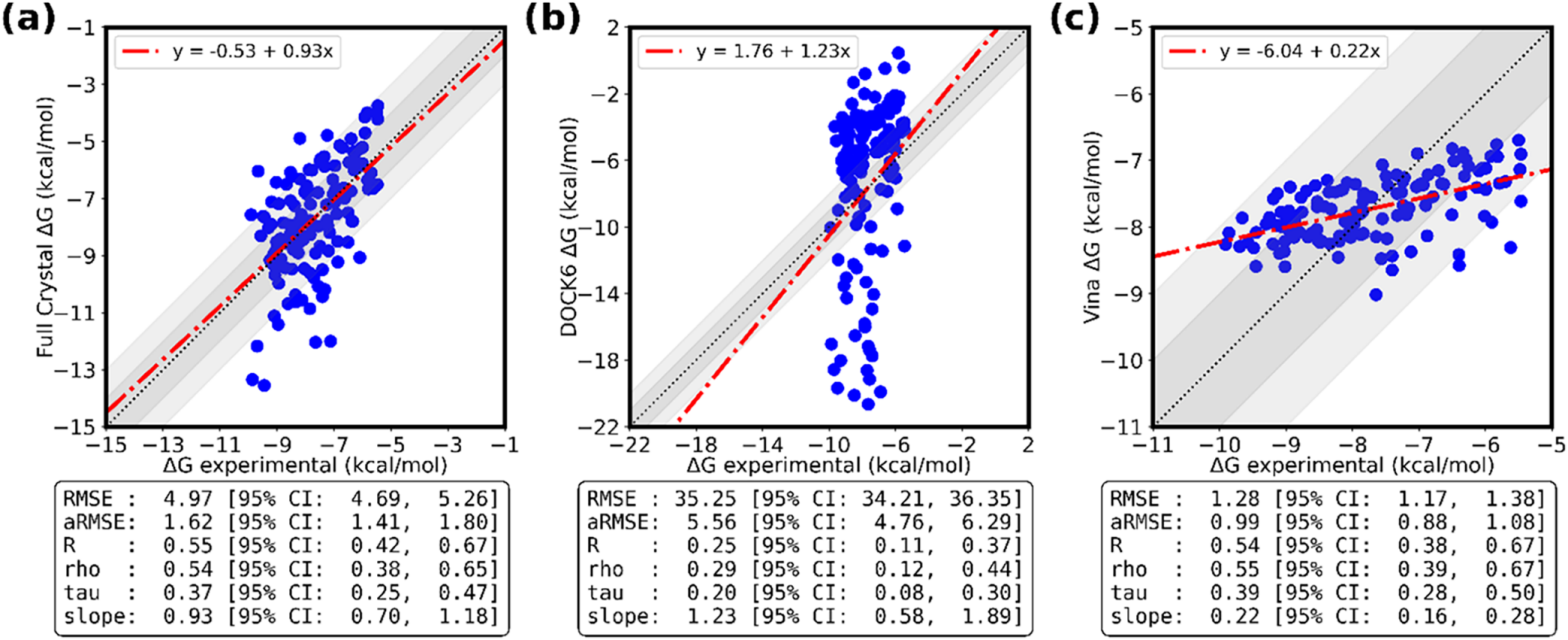
Comparisons of 130 computed (*y* axis) and experimental Δ*G* (*x* axis). (a) Δ*G* were computed using eq 3 with the Full Crystal scheme and a threshold of δ = 15 kcal/mol. (b) Δ*G* are molecular docking scores from DOCK6. Predictions have been shifted by their average offset from the experiment. The dark gray and light gray bands indicate ±1 kcal/mol and ±2 kcal/mol deviations from the diagonal, respectively, while the red dashed line represents the best-fit linear regression to the data. Performance metrics with 95% confidence intervals (CI) are shown in the table below the plot.

The improved accuracy and dynamic range of AlGDock compared to those of molecular docking require considerable computational cost. On a per ligand basis, AlGDock calculations take about 2 orders of magnitude more compute time than DOCK6 or Vina. For benchmarking details, see ***Section B.2 of the*** Supporting Information. The simulation length was sufficient to estimate BPMFs with a standard deviation less than 1 kcal/mol across 5 replicates in 80% of 40 representative systems, as described in ***Section B.3 of the*** Supporting Information.

For the 110 small molecules for which FEP calculations were reported, AlGDock was less accurate than FEP. FEP achieved an RMSE of 5.17 kcal/mol but an aRMSE of only 1.00 kcal/mol, indicating strong agreement with experimental values after correction of systematic error (Figure 3a). FEP exhibited excellent ranking ability with a Pearson *R* of 0.83, Spearman ρ of 0.84, and Kendall τ of 0.68, which were the highest among all methods that we compared.

**Figure 3:**
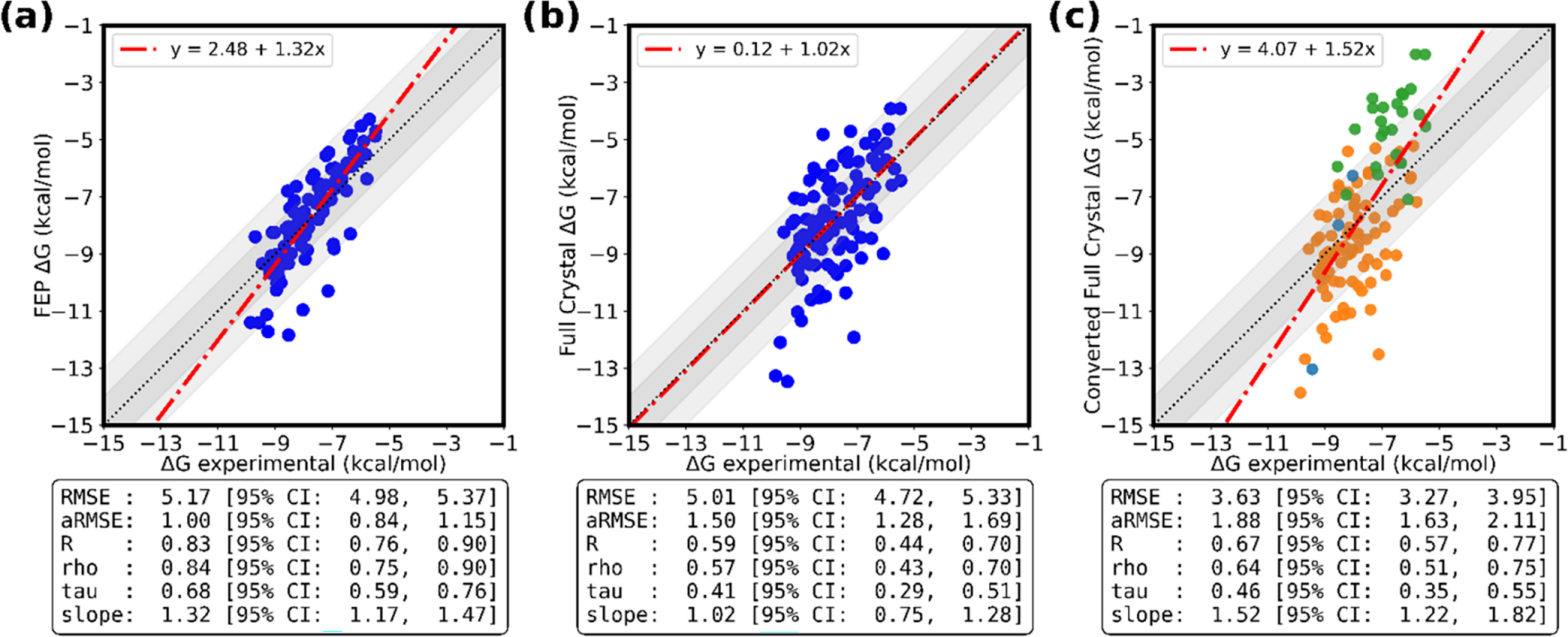
Δ*G* calculations (*y* axis) using (a) FEP, (b) Full Crystal scheme (eq 3), and (c) Converted Full Crystal scheme (eqs 4 and 5) applied to the set of 110 small molecules with available FEP data in comparison with experiment *x* axis). As in Figure 2, all predictions have been shifted by their average offset from the experiment. In panel (c), the data points are colored blue, orange, and green, corresponding to the reference ligands Mpro-P1800, Mpro-P0157, and Mpro-x10959, respectively. Statistical performance with 95% confidence intervals (CI) appears in the table below each plot. The dark gray and light gray bands represent ± 1 kcal/mol and ± 2 kcal/mol deviations from the regression line, respectively, while the red dashed line shows the ordinary least-squares linear regression fit for each panel.

In comparison, Full Crystal (Figure 3b) achieved a similar RMSE (5.01 kcal/mol) but a slightly higher aRMSE (1.50 kcal/mol). Its correlation values (*R* = 0.59, ρ = 0.57, τ = 0.41) were moderate; AlGDock did not match FEP’s ranking power. Compared to Full Crystal, Converted Full Crystal had a lower RMSE (3.63 kcal/mol) but a higher aRMSE (1.88 kcal/mol). Correlation values (*R* = 0.67, ρ = 0.64, τ = 0.46) were higher than Full Crystal but less than FEP. For both sets of estimates, the confidence intervals were about 0.05 wider than those from FEP. While Full Crystal outperformed other schemes, its success relies on crystal-specific information for snapshot selection. As detailed in ***Section B.4 of the*** Supporting Information, the inclusion of MSM weights did not significantly alter these outcomes. This indicates that the choice of snapshot selection strategy is a more critical determinant of Δ*G* accuracy than the MSM used for reweighting.

The relative performance of converted versus absolute Δ*G* calculations was dependent on the opposing factors of error cancellation and propagation. Higher correlation metrics of the converted calculations can be attributed to the cancellation of errors. On the other hand, the higher aRMSE and larger slope (Figures 3c, S4) can be attributed to error propagation. Among the three reference compounds, Δ*G* of Mpro-x10959 was anomalously low, leading to higher estimated ΔΔ*G* and overestimates of Δ*G*. Because this reference compound had a higher Δ*G* than the others, the slope increased.

We also evaluated the ability of different methods to classify ligands as tighter or weaker binders than those of another ligand (Figure 4). FEP provides the highest overall predictive accuracy (AUC = 0.89; logAUC = 0.38). While Full crystal has the same overall classification performance as Vina, its performance is superior for the tightest binders, where Vina performs comparably to random classification. Full Crystal significantly outperforms DOCK6 by both the AUC and logAUC metrics.

**Figure 4:**
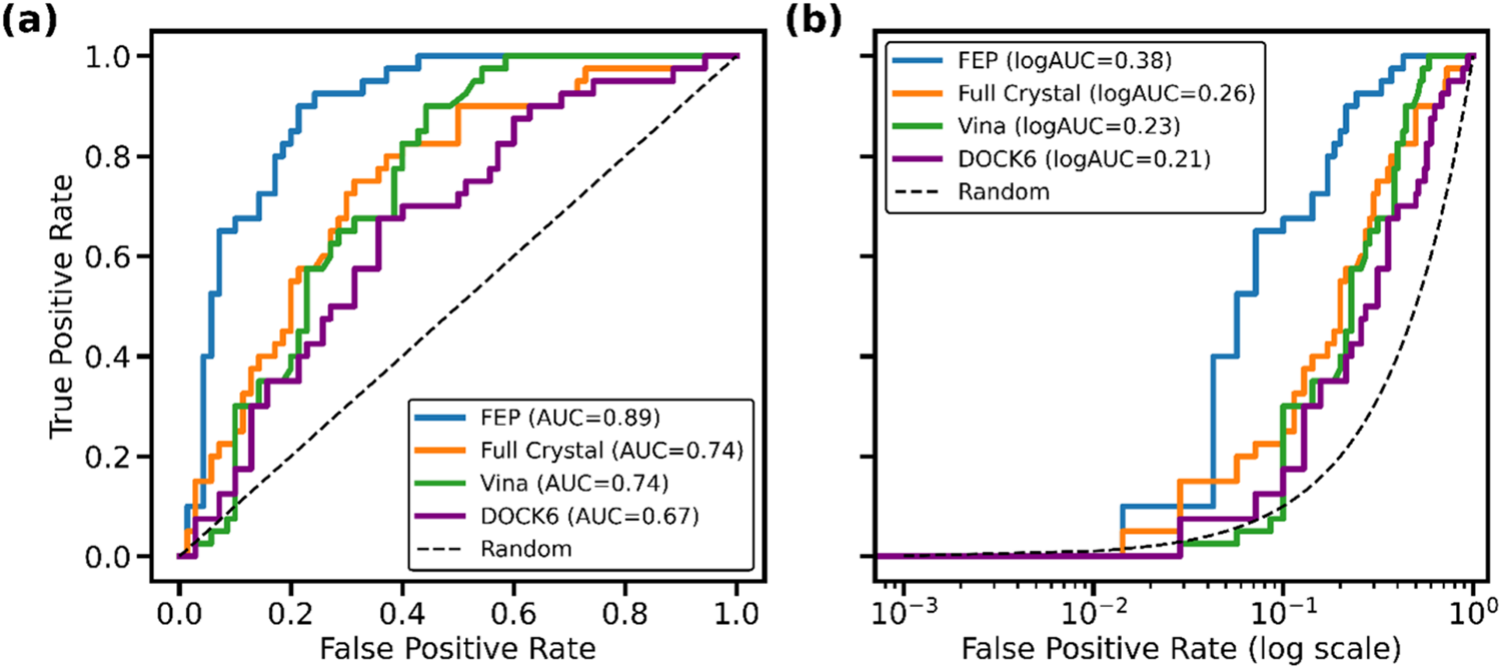
ROC analysis of screening performance and early enrichment. (a) ROC curve measures global classification accuracy. Ligands were categorized as stronger binders using a binary threshold defined by the mean experimental Δ*G* across all systems. (b) Semilog ROC plot highlights early enrichment by scaling the False Positive Rate (FPR) logarithmically from 0.001 to 1.

### AlGDock Predicted Native Poses with a Comparable Or Higher Success Rate and Fewer Decoys than Molecular Docking

We assessed pose prediction by the fraction of systems in which the native pose (RMSD < 3 Å) had a low score or ranked among the top 5 or 10 poses. This RMSD threshold for native poses is higher than the more commonly used cutoff of 2 Å, but the MPro catalytic site is larger and more solvent-exposed than most ligand binding sites, and COVID Moonshot drug leads are more flexible than most small-molecule ligands. For these complex ligands, predicted poses with RMSD values between 2 and 3 Å still position functional groups in the same direction and with similar orientations as crystallographic poses (Figure S11).

In a large majority of systems, AlGDock successfully scored the native pose as having a *similar* energy as the top pose (within 1 *k*_B_ *T*). In general, Full Crystal outperformed Docking and Random Crystal receptor snapshot selection schemes. All combinations of AlGDock snapshot selection and ranking schemes identified native poses within 6, 4, and 2 *k*_B_ *T* of the minimum with a success rate of at least 95%, 87%, and 77%, respectively (Table 1). However, the identification of native poses as the top-scoring poses (energy threshold of 0 *k*_B_ *T*) occurred in less than 40% of systems. Thus, there were many systems in which the native pose was sampled but outranked by geometric decoys with similar predicted free energies. Across snapshot selection schemes, the Receptor-weighted Pose BPMF ranking method achieved slightly higher success rates than the Pose Δ*G* at the high energy thresholds (Table 1). Both ranking approaches yielded the same rate of identifying the native pose as the top-scoring pose. Discrepancies between pose prediction performance with the Receptor-weighted Pose BPMF and the Pose Δ*G* are due to native subposes being clustered into a pose that is *not* represented by a native ligand snapshot.

**Table 1:** Fraction of Systems (Out of 130 Total Systems) in Which a Native Pose (RMSD < 3 Å from the Crystal Structure) Was Found within a Specified Energy Threshold (in *k*_B_ *T* Units) of the Lowest-Energy Snapshot: Docking Was Performed Using AlGDock, DOCK6, and Vina; Uncertainties Were Based on Bootstrapping 10,000 times with the Number of Systems Where Native Pose Was Found within the Specified Energy Threshold

Native within energy of minimum (k_B_T)	6	4	2	1	0
Scheme – Ranking type					
Full Crystal – Receptor-weighted Pose BPMF	0.97 ± 0.02	0.94 ± 0.02	0.92 ± 0.03	0.88 ± 0.03	0.40 ± 0.04
Full Crystal – Pose Δ*G*	0.96 ± 0.02	0.93 ± 0.02	0.89 ± 0.03	0.85 ± 0.03	0.40 ± 0.04
Random Crystal – Receptor-weighted Pose BPMF	0.99 ± 0.01	0.97 ± 0.02	0.92 ± 0.02	0.87 ± 0.03	0.33 ± 0.04
Random Crystal – Pose Δ*G*	0.99 ± 0.01	0.97 ± 0.02	0.92 ± 0.02	0.87 ± 0.03	0.33 ± 0.04
Docking – Receptor-weighted Pose BPMF	0.95 ± 0.02	0.90 ± 0.03	0.82 ± 0.03	0.78 ± 0.04	0.29 ± 0.04
Docking – Pose Δ*G*	0.95 ± 0.02	0.88 ± 0.03	0.78 ± 0.04	0.72 ± 0.04	0.29 ± 0.04
DOCK6 – Receptor-weighted Score	0.73 ± 0.04	0.54 ± 0.04	0.29 ± 0.04	0.20 ± 0.04	0.13 ± 0.03
DOCK6 – Pose Δ*G*	0.75 ± 0.04	0.51 ± 0.04	0.32 ± 0.04	0.18 ± 0.03	0.12 ± 0.03
Vina – Receptor-weighted Score	0.98 ± 0.01	0.97 ± 0.02	0.91 ± 0.03	0.82 ± 0.03	0.17 ± 0.03
Vina – Pose Δ*G*	0.95 ± 0.02	0.95 ± 0.02	0.87 ± 0.03	0.75 ± 0.04	0.21 ± 0.04

In terms of scoring the native pose with an energy similar to that of the top pose, AlGDock outperformed DOCK6 and Vina. As the Full Crystal and Random Crystal schemes incorporate at least some crystallographic ligand binding poses in BPMF calculations and receptor snapshot selection, it is most fair to compare *de novo* docking methods with the docking scheme. The docking scheme outperforms both DOCK6 and Vina at ranking the native pose as the top pose. At all other thresholds, the docking scheme based on the receptor-weighted pose BPMF achieved a significantly higher success rate than DOCK6 but the same or slightly lower success rate than Vina.

To address the concern that success in scoring the native pose may be achieved by assigning similar scores to many poses, we also considered whether the native pose was ranked among the top 1, 2, 5, or 10 poses (Table 2). The Pose Δ*G*, which reduces the number of poses by grouping subposes from different receptor conformations, excelled according to this metric. While the Full Crystal – Pose Δ*G* combination only identified the native pose as the top pose in 40% of systems, the fraction substantially increased when considering the Top 5 (70%) or Top 10 (80%). For all AlGDock calculations, the success rate for the Pose Δ*G* was higher than for the Receptor-weighted Pose BPMF. Among methods that do not use crystallographic ligand binding poses, the Docking – Pose Δ*G* outperforms DOCK6 and Vina for ranking the native pose in the Top 1 or 2, but DOCK6 and Vina scores (without clustering poses from different receptor conformations) have higher success rates for ranking the native pose in the Top 5 and 10.

**Table 2:** Fraction of Systems in Which the Native Pose (RMSD < 3 Å) Is Ranked within the Top 10, 5, 2, and 1 Predicted Poses Using Either the Receptor-Weighted Pose BPMF or Pose Δ*G* Ranking Protocols; Uncertainties Were Based on Bootstrapping 10,000 times with the Number of Systems Where Native Pose Was Found within the Specified Top N

scheme	ranking type	Top 10	Top 5	Top 2	Top 1
Full Crystal	Pose Δ*G*	0.80 ± 0.04	0.70 ± 0.04	0.52 ± 0.04	0.40 ± 0.04
	Receptor-weighted Pose BPMF	0.71 ± 0.04	0.58 ± 0.04	0.48 ± 0.04	0.40 ± 0.04
Random Crystal	Pose Δ*G*	0.77 ± 0.04	0.63 ± 0.04	0.50 ± 0.04	0.33 ± 0.04
	Receptor-weighted Pose BPMF	0.73 ± 0.04	0.60 ± 0.04	0.44 ± 0.04	0.33 ± 0.04
Docking	Pose Δ*G*	0.74 ± 0.04	0.55 ± 0.04	0.42 ± 0.04	0.29 ± 0.04
	Receptor-weighted Pose BPMF	0.61 ± 0.04	0.50 ± 0.04	0.37 ± 0.04	0.29 ± 0.04
DOCK6	Pose Δ*G*	0.83 ± 0.03	0.61 ± 0.04	0.28 ± 0.04	0.12 ± 0.03
	Receptor-weighted Score	0.77 ± 0.04	0.52 ± 0.04	0.25 ± 0.04	0.13 ± 0.03
Vina	Pose Δ*G*	0.74 ± 0.04	0.62 ± 0.04	0.38 ± 0.04	0.21 ± 0.04
	Receptor-weighted Score	0.75 ± 0.04	0.61 ± 0.04	0.32 ± 0.04	0.17 ± 0.03

The distance threshold used in clustering subposes affects ranking performance (Table S3). As the distance threshold increases, the success rates for identifying native poses within the top 10 and top 5 improved markedly. This trend can be attributed to the fact that larger thresholds produce fewer but more populated clusters, increasing the likelihood that a cluster represented by the native pose will appear among the top-ranked groups. However, the top 1 success rate remained constant across all thresholds.

While AlGDock is able to recapitulate major structure–activity relationships (***section B.5 of the*** Supporting Information), at least some AlGDock free energy and pose prediction failures stem from the heuristic treatment of protein flexibility and the use of implicit solvent. In one illustrative system with a high aRMSE (Figure S12), the ligand contains both a polar sulfonyl group and a nonpolar cyclobutanyl group. In the crystallographic binding pose, the sulfonyl group is oriented toward a nearby water molecule, suggesting a hydrogen-bonding interaction, while the cyclobutyl group is exposed to bulk solvent and unobstructed by binding site residues. In contrast, the predicted pose places both groups inward toward the protein, where the cyclobutyl region is partially obstructed by surrounding side chains. While principal component analysis suggested that Folding@home trajectories are a superset of MPro conformations, the presence of obstructing side chains shows that the analysis does not account for more subtle structural rearrangements. Moreover, an implicit solvent would not incorporate the hydrogen bond between the sulfonyl group and the stable water observed in the crystal structure.

### Successful Pose Prediction Promotes but is Not Required for Accurate Δ*G* Calculations

Across the three schemes (Docking, Full Crystal, and Random Crystal), the systems in which the native pose is ranked as the top candidate exhibit relatively narrow and centered error distributions (Figure 5a), indicating that selecting the correct pose generally leads to smaller deviations between predicted and experimental Δ*G* values. The aRMSE values for systems that successfully identified the native pose as the top-scoring configuration were 1.33, 1.66, and 1.88 kcal/mol for Full Crystal, Random Crystal, and Docking, respectively. When the difference between the native and top-scoring pose was within 3 kcal/mol, the aRMSE increased slightly to 1.64, 1.85, and 1.85 kcal/mol for the three schemes. When this difference exceeded 3 kcal/mol, the aRMSE rose substantially for Docking (3.15 kcal/mol) and moderately for Full Crystal (1.88 kcal/mol), while Random Crystal remained lower and relatively stable at around 1.8 kcal/mol (Figure 5b). This error is driven by decoy poses with much lower BPMFs than the native pose.

**Figure 5:**
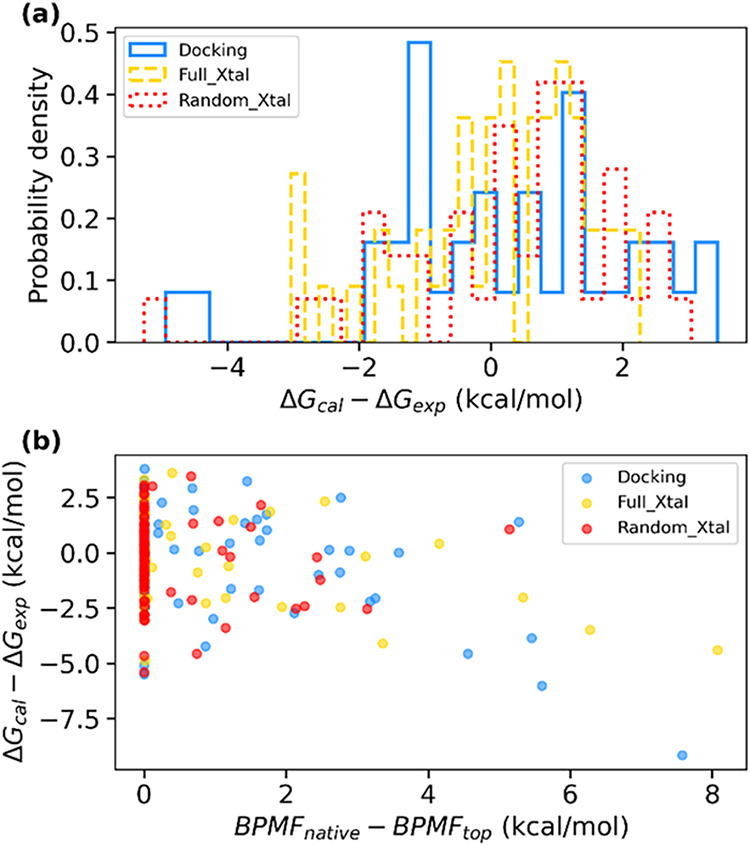
Relationship between pose prediction (Receptor-weighted Pose BPMF) and Δ*G* accuracy. (a) Distribution of Δ*G* prediction errors for systems in which the native pose is ranked as the top pose. (b) Dependence of Δ*G* prediction error on the deviation of the native pose from the top-ranked pose for systems where the native pose is not ranked first. The colors blue, yellow, and red correspond to Docking, Full Crystal, and Random Crystal schemes, respectively.

## Discussion

### Apo MDS Can Capture the Receptor Flexibility Needed for Accurate Δ*G* Calculations

The treatment of receptor flexibility remains a significant challenge in molecular docking. While calculations that allow ligand flexibility are routine, permitting receptor flexibility can introduce too many variables that lead to prohibitive computational costs. One strategy that has been developed to address this challenge is ensemble docking – the use of multiple high-resolution structures or MDS snapshots of a receptor.^27,28^ MDS can reveal cryptic pockets that are not visible in high-resolution structures.^33^

To our knowledge, we are the first to report Δ*G* calculations based on using BPMFs to apo MDS. The typical application of ensemble docking is for virtual screening to identify the likely hit compounds within a chemical library; it is seldom used for Δ*G* calculations. Smith et al.^54^ reported Δ*G* calculations based on ensemble docking to MSMs of apo T4 lysozyme L99A, but used docking scores in lieu of BPMFs. Our group’s previous work with T4 lysozyme L99A was based on holo MDS^24^ and in the Drug Design Data Resource Grand Challenge 3 (Subchallenge 2) was with holo crystallographic structures.^25^

In principle, differences between apo and holo ensembles can present a greater challenge to Δ*G* calculations based on ILT^20^ compared to the closely related parallelized-overparts free energy perturbation (POPFEP)^55^ framework. In POPFEP, configuration space is partitioned into mutually exclusive regions. Regional free energy differences (e.g., for ligand decoupling) are computed independently for each region and combined based on the equilibrium probability of each region in an initial thermodynamic state (e.g., the apo state). As it only requires sampling within a region, calculating regional free energy differences should incur less computational cost than calculating global free energy differences. ILT^20^ can be thought of as a specific case of POPFEP^55^ in which the region is defined by a completely rigid receptor snapshot. Rigidifying the receptor enables computational speedups (e.g., interpolation of precomputed receptor–ligand interaction energies^23^) at the cost of failing to capture subtle receptor changes that are available to simulations with a fully flexible receptor.

Our results here demonstrate that apo MDS can mostly capture the receptor flexibility needed for accurate Δ*G* calculations. While principal components analysis suggested that apo MDS are a superset of the holo ensemble of MPro,^35^ it was not clear whether apo receptor configurations are similar enough to holo configurations for accurate Δ*G* calculations. MPro is a flexible protein with a solvent-exposed binding site that can accommodate a range of peptide substrates.^35^ Moreover, the ligand library did not comprise small and rigid fragments, but lead- or drug-like molecules. Despite these challenges, AlGDock was much more accurate at computing Δ*G* than DOCK6 and approached the accuracy of FEP. Previous studies have shown that docking scores have poor correlation with experimental binding free energies,^56,57^ but FEP can achieve high accuracy.^16,58,59^ Correlation metrics here were comparable to the best performing AlGDock calculations in the Drug Design Data Resource Grand Challenge 3 (Subchallenge 2); for ligands binding to the vascular endothelial growth factor receptor 2, Δ*G* calculations with the Full solvation option obtained a Pearson R of 0.49 (0.13), a Spearman ρ of 0.42 (0.14), and a Kendall τ of 0.3 (0.14) in comparison with experimental values.^25^

As a caveat, the ability of apo MDS to capture receptor flexibility is system-dependent. As we observed in this study, even subtle changes in the receptor conformation that are unique to a specific ligand can lead to inaccurate Δ*G* calculations. If apo and holo ensembles have very distinct binding site conformations, another receptor sampling strategy may be required. If receptors are sampled from a holo ensemble, ILT may be directly used to compute relative free energies (without the conversion performed in this project).^21,22^

### MSMs Provide a Principled Way to Weight Receptor Conformations for Ensemble Docking

Several studies have demonstrated the suitability and benefits of using MSMs in ensemble docking. Mukherjee et al.^60^ performed MDS of the apo mouse double minute 2 homologue, constructed an MSM, and docked known ligands to MSM microstates. The success rate of ligand pose prediction was comparable to cross-docking (docking ligands to receptor structures obtained in complex with other ligands). Kamenik et al.^61^ performed MDS and constructed an MSM of T4 lysozyme L99A. While standard docking scores yielded many decoys, adding energetic penalties based on MSM probabilities (with an empirical scaling factor) led to improved enrichment of known ligands. Smith et al.^54^ also performed MDS and constructed an MSM of T4 Lysozyme L99A. Subsequently, they docked 17 ligands to representative snapshots from each MSM microstate. Δ*G* calculations performed with a MSM-weighted exponential average of docking scores outperformed those based on the best docking score.

While our MSM-derived weights were nearly uniform across binding site conformations, this is generally not the case. Mukherjee et al.^60^ observed that most favorable docking scores were obtained for closed conformations (Figure 5 of their paper) with a wide range of equilibrium probabilities (Figure 2 of their paper). Kamenik et al.^61^ found distinct equilibrium probabilities for three binding pocket conformations: open (0.16%), intermediate (1.09%), and closed (98.76%). Smith et al.^54^ observed that the χ_1_ torsion angle distribution of Valine 111, which lines the artificial binding pocket created by the L99A mutation, has multiple peaks of varying population that largely collapse to one upon ligand binding.

The benefit of incorporating MSM-derived weights into ensemble docking is to account for receptor reorganization free energies, which can be a major factor in molecular recognition processes.^62^ For example, the selectivity of imatinib mesylate for Abl tyrosine kinase over closely homologous proteins such as c-Src has been attributed in large part to the greater reorganization free energy of the DFG loop in the latter.^63^ The higher potency of the second-generation inhibitor dasatinib may be largely due to avoiding the reorganization free energy of another motif, the glycine-rich P-loop.^64^ These contrasting case studies from different parts of the same binding pocket illustrate the potential importance of incorporating receptor reorganization into Δ*G* calculations.

### Ligand Reorganization Can Be a Major Factor in the Accuracy of Δ*G* Calculations

While our calculations did not illustrate the importance of receptor organization, they did demonstrate the importance of ligand reorganization. Although several studies have used MSMs for ensemble docking, to our knowledge, our present work is the first to use BPMFs (opposed to docking scores) within MSM-based Δ*G* calculations. BPMFs mainly differ from docking scores by including ligand reorganization free energies due to changes in configurational entropy in addition to interaction energies. The fact that our Δ*G* calculations based on BPMFs were much more accurate than those based on docking scores (Figure 2) highlights the importance of this factor.

Given the importance of ligand reorganization, it is tempting to attribute the relatively poor performance of Δ*G* calculations in Smith et al.^54^ to the use of interaction energies opposed to BPMFs. Notwithstanding the vastly different receptor and ligands, the Pearson R of 0.281 reported by Smith et al.^54^ is strikingly comparable to ours from calculations based on docking scores (Figure 2 ***b***). However, in another study of T4 lysozyme binding to fragment-like ligands, correlation metrics only improved modestly from a Pearson R of 0.50 (0.04) using DOCK6 scores to 0.65 (0.05) using BPMF calculations (Table 3 of the Xie, Nguyen, and Minh).^24^

The relative importance of ligand reorganization is dependent on the complexity of the ligand. While the ligands from the T4 lysozyme L99A studies^24,54^ were relatively small and rigid, the ligands in this present study are more drug-like and complex, with a higher molecular weight and more rotatable bonds. The restriction of rotatable bonds when a ligand transitions from solution to a protein complex reduces its configurational entropy. This configurational entropy loss is caused by the narrowing of energy wells in the bound state and has no observable correlation with the rotatable bond count.^65,66^ Thus, the accurate Δ*G* calculations in this study required the use of statistically rigorous methods^23^ to compute ligand reorganization free energies due to the narrowing of these wells.

In addition to ΔG, ligand reorganization can affect binding poses. For example, Xie et al.^67^ identified two binding poses of fentanyl and its analogues in the μ opioid receptor that have similar mean interaction energies. By analyzing the energy landscapes of the unbound and bound ligands, Minh, Cooper, Xie, and Shi (2025)^68^ found that the ligand conformation determined by cryo-electron microscopy was preferred due to a lower ligand reorganization free energy; this pose has a lower free energy in the apo landscape.

### Implicit Solvent Could Limit the Accuracy of AlGDock

While our workflow is based on rigorous treatments of receptor and ligand reorganization, its accuracy may be limited by the solvent model. We used the Onufriev, Bashford, and Case model 2 (OBC2)^69^ implicit solvent model, which treats solvent as a continuum with a high dielectric constant. It does not explicitly represent hydrogen bonding or water-mediated stabilization. These limitations could affect binding poses and affinities, especially for ligands that extend into solvent-exposed regions or interact with bulk water or other miscible solvents.^70^ MPro has an open and solvent-exposed site.^71^ The solvent model could be one of the reasons that our present Δ*G* calculations have lower correlations with experiment than our group’s previous calculations with T4 lysozyme,^24^ where the binding site is more buried, concave, and nonpolar.^72^

### Clustering Groups Together Decoys to Improve the Rank of Native Poses

We have invented an approach to predict binding poses using multiple receptor snapshots. Calculating binding poses based on the lowest interaction energy neglects entropy. The Pose BPMF^23^ accounts for loss in the configurational entropy of the ligand, but not the receptor. The intent of our new approach, the Pose Δ*G*, was to account for configurational entropy of the receptor by favoring ligand binding poses that appear in multiple receptor conformations.

Contrary to our expectations, the Pose Δ*G* approach did not lower the free energy of the native pose as much as it grouped together decoys to reduce the number of poses. The Pose Δ*G* ranked the native pose as top at the same rate as the Receptor-weighted Pose BPMF (Table 1). The lack of improvement suggests that for the selected systems, the binding pose is dominated by a single receptor conformation. Indeed, due to grouping native subposes into poses that are *not* represented by a native ligand snapshot, the Pose Δ*G* method reduced success rates at thresholds of 1, 2, 4, and 6 *k*_B_ *T* (Table 1). On the other hand, the Pose Δ*G* was more successful than the Receptor-weighted Pose BPMF at ranking the native pose in the top 5 and 10 (Table 2). The increased success of the top N ranking is due to a reduced number of decoy poses. In summary, clustering ligand snapshots across receptor snapshots can be thought of as an imperfect filter of decoy poses.

The low success rates of all methods in pose prediction reflect the difficulty of the problem. AlGDock is capable of self-docking 92.9%^23^ of the Astex Diverse Set,^73^ which comprises ligands of variable complexity in high-quality binding poses. The process of selecting systems with high-quality poses favors rigid complexes. Here, we have attempted cross-docking to multiple receptor snapshots. While crystal structures of the complexes have been solved, we did not use any quality filters that could limit problem complexity.

### Using Experimental Structures to Filter Decoys Can Improve the Accuracy of AlGDock

While crystallographic structures were available for all of the protein–ligand complexes in this study, we anticipate that in drug discovery settings, this high-information scenario will be rare. Thus, we evaluated three snapshot selection schemes that use differing amounts of structural data: Docking, which does not use any experimental structures; Random Crystal, which uses receptor structures for a subset of ligands; and Full Crystal, which uses complete structures for all ligands. Unsurprisingly, incorporating more structural data led to more accurate Δ*G* calculations and pose predictions.

While our calculations demonstrated the benefits of using experimental structures, results with the Random Crystal scheme suggest that these data have diminishing returns. The Random Crystal approach achieved performance comparable to Full Crystal while not requiring high-resolution structures of each receptor–ligand pair (Figures S4, S6b, S8c, S8d). Performance gradually improves with the number of crystal structures, but beyond 12 crystal structures, correlation and error metrics did not significantly improve (Figure S7). The effectiveness of using a small subset of crystals could arise from the consistency between binding pockets across ligands,^74^ enabling accurate calculations even in the absence of full crystallographic coverage. In general, the precise number of crystal structures necessary for accurate Δ*G* calculations is likely to depend on the complexity of the binding site and the structural diversity of receptor–ligand interactions.

The different snapshots used for BPMF estimation led to distinct sampling of predicted ligand poses across the schemes. Even when considering only Random Crystal and Docking, both of which exclude ligand crystal poses during BPMF estimations, Random Crystal still achieves better pose prediction than Docking (Tables 1 and 2). This highlights that the sampling of ligand poses is strongly influenced by the underlying binding site geometries and their associated weights. The choice of snapshots therefore affects pose sampling, pose ranking, and ultimately Δ*G* accuracy.

Correct ranking of the native pose is helpful but not required for accurate Δ*G* calculations. When the native pose is correctly ranked as the top pose, the aRMSE can be as low as 1.3 kcal/mol. Decoys, snapshots that the force field assigns a lower free energy than the native pose, degrade the accuracy of AlGDock. Contrasting the performance of receptor snapshot selection schemes indicates that the receptor snapshot has a larger influence on Δ*G* accuracy than the ligand snapshot. Even with large differences in pose BPMFs, decoy poses that bind to the native receptor snapshots (as in the Full and Random Crystal scheme) increase the aRMSE, but only to around 1.8 kcal/mol. In many of these cases, the native pose has an erroneously high BPMF (Figure 5b), possibly because of the limited flexibility of receptor conformations. The relatively low aRMSE is possible because decoy poses bind with a similar free energy as the native pose should. However, if decoy poses bind to non-native receptor snapshots (which can occur in the Docking scheme), then calculated Δ*G* can be significantly lower than experiment (Figure 5b). These decoy poses explain the overall performance differences between the Docking and Random Crystal scheme; the latter scheme filters out receptor snapshots that do not accommodate the native ligand binding pose.

The fact that using experimental structures to filter out decoys improves the accuracy of Δ*G* calculations has broader implications for the selection of receptor configurations in ensemble docking. When many receptor configurations are available, a critical decision in ensemble docking lies in determining which to select for molecular docking (or BPMF) calculations. Our previous work on the efficiency of stratification^26^ and in the Drug Design Data Resource Grand Challenge 3 ^75^ demonstrated the importance of selecting diverse receptor conformations. The work reported here suggests that it is also beneficial to select receptor conformations that strongly interact with the native binding pose of the ligand, as opposed to alternative conformations that bind to decoys.

### AlGDock Requires More Overhead But Less Compute Time Per Ligand than FEP

Given that the accuracy of AlGDock rivals FEP, it is worth discussing its relative computational cost. Factors that affect the relative computational cost include the size and complexity of receptors and ligands, and the number of ligands. In contrast to FEP, AlGDock requires an initial overhead cost in sampling receptor conformations and computing their equilibrium probabilities. If these calculations have already been performed to understand the dynamics of the apo receptor (as in the initial calculations for this study^33,35^ ), AlGDock provides an opportunity to repurpose them for Δ*G* calculations. Even if the benefits of understanding receptor dynamics are not considered, however, the computational investment in receptor sampling can pay off. These benefits are more substantial if the receptor is large or has slow conformational dynamics. Precomputing dynamics is helpful for large receptors because AlGDock primarily calculates receptor–ligand interactions using grid interpolation,^23,76^ which scales nearly linearly with ligand, as opposed to molecular system size (with a neighbor list). It is helpful for slow dynamics because, otherwise, these motions would need to be sampled in every complex. Precomputing receptor dynamics is most advantageous for performing Δ*G* calculations for a large library of smaller ligands. For a large library, the cost per ligand is lower. In contrast to larger ligands, small ligands have lower costs for grid interpolation and involve a smaller fraction of energy terms; there is a larger benefit to skipping energy terms that exclude the ligand.

For the systems in this study, AlGDock required significantly less computational expense per ligand than FEP. Each FEP transformation (ΔΔ*G*) required approximately 2–4 GPU-days on high-performance GPUs such as NVIDIA V100s.^16^ Based on reported GPU-to-CPU throughput ratios (Hess et al.),^77^ this translates to roughly 1920–3840 CPU-core hours per transformation. In contrast, BPMF calculations require 10–24 h of CPU time on a single core (see ***Section B.2 of the*** Supporting Information). To compute BPMFs for 30–40 rigid receptor snapshots, 300–960 CPU-core hours are required per ligand, which is considerably less than FEP. As an additional benefit, BPMF calculations are fully independent and can be efficiently distributed across CPU resources, allowing many Δ*G* calculations to be completed within a day using high-throughput computing. In future work, GPU acceleration of AlGDock simulations may further improve computational efficiency.

### AlGDock Can Be a Suitable Tool for Early-Stage Drug Discovery

Through this project, we have significantly advanced AlGDock toward applications in drug discovery. Our group’s earliest work on AlGDock applied it to the binding of fragment-like molecules to a simple model binding site.^24^ Later work considered more complex ligands and receptors, but did not use adequate receptor sampling and snapshot selection strategies.^75^ Here, we have demonstrated that extensive molecular dynamics simulations of the apo ensemble and appropriate snapshot selection strategies can adequately represent the holo ensemble of a flexible protein, SARS-CoV-2 MPro, to accurately compute Δ*G* for drug lead compounds. AlGDock can leverage a theoretically rigorous treatment of receptor and ligand organization free energies to perform Δ*G* calculations that outperform docking scores and rival the accuracy of FEP across a broad dynamic range.

While AlGDock and Vina demonstrated comparable ranking power (τ = 0.37 vs 0.39) (Figure 2b,c), the more accurate dynamic range of AlGDock is critical for secondary virtual screening applications. Vina is one of the most widely used molecular docking programs and a common starting point for virtual screening campaigns.^78,79^ However, our observation of a shallow slope (0.22) (Figure 2 ***c***) suggests that it lacks the sensitivity required to provide clear separation of active and inactive compounds. The limited sensitivity of Vina is also supported by ROC analysis of its ability to categorize ligands as tighter or weaker binders (Figure 4). Although its global AUC is equal to AlGDock, its ability to classify the *top-ranked compounds* is comparable to random. This limitation justifies a hierarchical workflow in which the speed of docking is leveraged in a primary virtual screen to filter large libraries, followed by a secondary virtual screen with a more physically rigorous method to more accurately prioritize a subset.^19^ AlGDock produced a near-unit regression slope (0.93) (Figure 2b) and a higher logAUC (0.26) than Vina, demonstrating superior sensitivity to the true magnitude of binding and suitability to fill this niche.

The requirement of suitable training data limits the potential of machine learning methods in secondary virtual screening applications. Machine learning methods have demonstrated promising results in predicting binding affinities at reduced computational cost,^80^ and their potential in drug discovery is widely acknowledged. However, their accuracy is highly dependent on the quality and diversity of training data, with performance dropping substantially when proper cross-validation schemes are applied to novel targets.^81,82^ In this context, physics-based methods such as AlGDock offer a complementary strategy that does not rely on training data, making them more suitable for prioritizing structurally diverse analogues in virtual screening applications.

A confluence of factors suggests that early-stage drug discovery would leverage the advantages and mitigate the disadvantages of AlGDock relative to FEP. Compared to lead optimization, secondary virtual screening and hit optimization involve larger libraries of smaller compounds. The combination of high overhead costs and lower costs per additional ligand is most advantageous for screening large libraries of compounds. Interpolation of precomputed receptor–ligand interaction energies is most beneficial for smaller ligands. When hits are obtained, there may be limited experimental information about their binding poses. The ability to readily incorporate multiple binding poses into a BPMF calculation^23^ is most beneficial when the binding pose is unknown. Regarding the mitigation of disadvantages, the limitations of AlGDock in sampling more subtle receptor rearrangements and modeling solvent interactions are less relevant in early-stage discovery. The primary consequences of these failures are to overestimate Δ*G* and produce false negatives. As the primary goal of early-stage drug discovery is to identify hits and prioritize the most promising compounds, it is more important to filter false positives that increase the cost of additional experiments than to eliminate false negatives. Thus, AlGDock is well-suited for secondary virtual screening that can bridge the gap between molecular docking and FEP or experiment.

## 5 Conclusions

In this work, we estimated the binding free energies of 130 small molecules from the COVID Moonshot project^16^ using AlGDock and compared the results to DOCK6, Vina, and experimental data. For 110 of these molecules, comparisons were also made with FEP calculations. AlGDock demonstrated moderate accuracy in predicting absolute binding free energies, with correlation metrics of *R* = 0.67, ρ = 0.64, and τ = 0.46, compared to FEP’s higher accuracy (*R* = 0.83, ρ = 0.84, τ = 0.68). However, AlGDock required significantly less computational resources than FEP.

AlGDock outperformed molecular docking methods in the pose prediction. While DOCK6 and Vina were successful in ranking the native pose as the top pose in only about 20% of the 130 systems, AlGDock had a higher success rate of 30–40%. However, AlGDock, DOCK6, and Vina had similar success rates of obtaining native poses within the top 5 and 10 predicted poses.

These findings highlight AlGDock’s potential as a scalable and computationally affordable tool for secondary screening of small-molecule libraries in early-stage drug discovery.

## Supplementary Material



## Data Availability

BPMF calculations were performed using AlGDock, which is available at https://github.com/CCBatIIT/AlGDock. Input files, including 3D structures of ligands, protein conformations, and custom scripts that we used for this manuscript, are available on GitHub (https://github.com/EllaNguyen1711/ABFE). The code/bash scripts have been organized and numbered to match the intended order of execution.

## Notes

Chemical structures, SMILES strings, and free energy data of the 130 compounds. Supporting Results about MSM construction, AlGDock computational cost, structure–activity relationships, and factors affecting AlGDock accuracy. Additional figures with receptor weights and illustrative comparisons between computed and crystallographic poses. A table demonstrating the effects of varying a threshold. A figure showing the cause of an AlGDock failure (PDF)
