# Supplementary material for: Absolute Binding Free Energy Calculations between the SARS-CoV‑2 Main Protease and 130 Drug Leads Using Implicit Ligand Theory
Source: J Chem Inf Model. 2026 May 20;66(11):6576–90. doi: 10.1021/acs.jcim.6c00077 (PMC13250917; doi:10.1021/acs.jcim.6c00077)
Supplement: Supplementary file 1 [file ci6c00077_si_001.pdf]

SUPPORTING INFORMATION

Absolute Binding Free Energy Calculations between the SARS-CoV-2 Main Protease and 130 Drug Leads using Implicit Ligand Theory

Hong Ha Nguyen, Bing Xie, and David D. L. Minh*

Department of Chemistry, Illinois Institute of Technology, Chicago, IL 60616, USA

*Corresponding author. Email: dminh@illinoistech.edu

Contents

A Supporting Material 3

Table S1. Small molecules from the COVID Moonshot used in this study 3

B Supporting Results 18

B.1 Density-based clustering yields similar kinetics as K-means clustering with fewer microstates 18

Figure S1. Implied timescale analysis used to select the MSM lag time. 18

Figure S2. Chapman–Kolmogorov (CK) test for the five-state Markov state model at a lag time of 30 steps (3 ns). 19

B.2 The computational cost of AlGDock is much more than molecular docking 20

B.3 BPMF calculations are converged 20

Figure S3. Ensemble convergence behavior during interaction grid scaling (CD). 20

B.4 The accuracy of ΔG calculations depended on the threshold energy and snapshot selection scheme, but not MSM weights 20

Figure S4. Performance of ΔG calculations across different snapshot selection schemes as a function of threshold (δ). 21

Figure S5. Distributions of selected snapshot counts across energy thresholds (δ) for the three schemes. 22

Figure S6. Alchemical free-energy predictions using a) Docking and b) Random Crystal scheme (AlGDock). 23

Figure S7. Correlation metrics as a function of the number of crystal structures. 23
Figure S8. Alchemical free-energy predictions using (a) Docking, (b) Converted Docking, (c) Random Crystal and (d) Converted Random Crystal. 24
Table S2. Effects of MSM weights on exponential averages of BPMFs. 25
Figure S9. Histograms of weights for receptor states. 26
B.5 AlGDock recapitulates major structure-activity relationships 27
Figure S10. 28
Figure S11. Comparison of predicted and crystallographic ligand poses across different RMSD ranges. 29
Table S3. Comparison of the success rates in predicting native poses among three schemes. 30
Figure S12. Example of a pose prediction failure for a ligand with a large binding free energy error. 31
C References 32

A Supporting Material

Table S1. Small molecules from the COVID Moonshot used in this study. This table includes the following columns: crystal name, Moonshot ID, 2D structure, experimental ΔG (kcal/mol), FEP estimated ΔG (kcal/mol) by the COVID Moonshot project (if available), batch ID (sprint) for small molecule submission (if available), and SMILES notation for each small molecule.

*ref is the reference compound used for FEP estimation for all small molecules within the same batch (sprint).

**NaN values are not available

	NAME CRYSTAL	CID	Structure	EXPT ΔG	FEP ΔG	sprint	SMILES
1	Mpro-P2263	EDJ-MED-8bb691af- 6	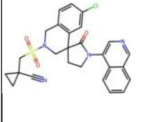	-9.45	-14.42	11A	c1ccc2c(c1)cncc2N3CCC4(C3=O)CN(Cc5c4cc(cc5)Cl)S(=O)(=O)CC6(CC6)C#N
2	Mpro-P2017	MAT-POS-4223bc15-12	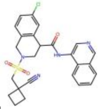	-9.86	-16.47	8	c1ccc2c(c1)cncc2NC(=O)C3CN(Cc4c3cc(cc4)Cl)S(=O)(=O)CC5(CCC5)C#N
3	Mpro-P1010	MAT-POS-4223bc15-18	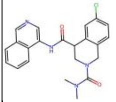	-8.96	-15.34	8	CN(C)C(=O)N1Cc2ccc(cc2C(C1)C(=O)Nc3cncc4c3cccc4)Cl
4	Mpro-P1090	MAT-POS-4223bc15-23	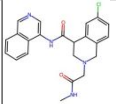	-9.70	-13.48	8	CNC(=O)CN1Cc2ccc(cc2C(C1)C(=O)Nc3cncc4c3cccc4)Cl
5	Mpro-P1202	MAT-POS-4223bc15-28	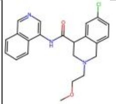	-7.71	-11.44	8	COCCN1Cc2ccc(cc2C(C1)C(=O)Nc3cncc4c3cccc4)Cl

6	Mpro-P0906	JIN-POS-6dc588a4- 6	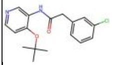	-6.34	-9.92	9	CC(C)(C)Oc1ccncc1NC(=O)Cc2cccc(c2)Cl
7	Mpro-P0851	MAT-POS-dd3ad2b5- 3	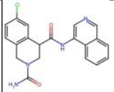	-9.01	-14.27	8	c1ccc2c(c1)cncc2NC(=O)C3CN(Cc4c3cc(cc4)Cl)C(=O)N
8	Mpro-P0151	EDG-MED-70ae9412-2	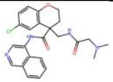	-7.24	-11.66	8	CN(C)CC(=O)NCC1(CCOc2c1cc(cc2)Cl)C(=O)Nc3cncc4c3cccc4
9	Mpro-P0224	EDJ-MED-8c98ee63- 2	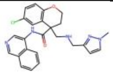	-8.32	-12.20	8	Cn1ccc(n1)CNCC2(CCOc3c2cc(cc3)Cl)C(=O)Nc4cncc5c4cccc5
10	Mpro-P0805	EDG-MED-ba1ac7b9- 21	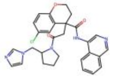	-8.48	-12.47	8	c1ccc2c(c1)cncc2NC(=O)C3(CCOc4c3cc(cc4)Cl)CC(=O)N5CCCC5Cn6ccnc6
11	Mpro-P0766	EDJ-MED-9e38fd34- 1	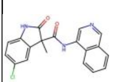	-8.49	-13.49	8	CC1(c2cc(ccc2NC1=O)Cl)C(=O)Nc3cncc4c3cccc4
12	Mpro-P0878	EDJ-MED-2f867453-1	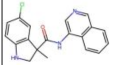	-7.96	-12.78	8	CC1(CNc2c1cc(cc2)Cl)C(=O)Nc3cncc4c3cccc4
13	Mpro-P0743	KAD-UNI-80f122c8-2	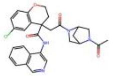	-7.40	-11.98	8	CC(=O)N1CC2CC1CN2C(=O)CC3(CCOc4c3cc(cc4)Cl)C(=O)Nc5cncc6c5cccc6
14	Mpro-P0074	RAL-THA-2d450e86-12	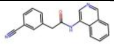	-7.84	-13.16	8	c1ccc2c(c1)cncc2NC(=O)Cc3cccc(c3)C#N

15	Mpro-P0075	RAL-THA-2d450e86-16	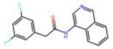	-8.53	-14.07	8	c1ccc2c(c1)cncc2NC(=O)Cc3cc(cc(c3)F)F
16	Mpro-P0063	RAL-THA-2d450e86-33	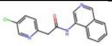	-6.71	-11.20	8	c1ccc2c(c1)cncc2NC(=O)Cc3ccc(cn3)Cl
17	Mpro-P0125	EDG-MED-971238d3-1	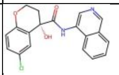	-8.20	-13.15	8	c1ccc2c(c1)cncc2NC(=O)[C@@]3(CCOc4c3cc(cc4)Cl)O
18	Mpro-P0060	MIC-UNK-45817b9b-1	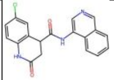	-8.67	-13.81	8	c1ccc2c(c1)cncc2NC(=O)C3CC(=O)Nc4c3cc(cc4)Cl
19	Mpro-P0122	RAL-THA-8416115c-5	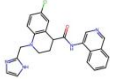	-8.34	-12.61	8	c1ccc2c(c1)cncc2NC(=O)C3CCN(c4c3cc(cc4)Cl)Cc5[nH]ccn5
20	Mpro-P0056	RAL-THA-4aa06b95- 1	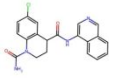	-8.19	-12.95	8	c1ccc2c(c1)cncc2NC(=O)C3CCN(c4c3cc(cc4)Cl)C(=O)N
21	Mpro-x12582	ERI-UCB-d6de1f3c- 2	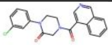	-6.88	-11.77	8	c1ccc2c(c1)cncc2C(=O)N3CCN(C(=O)C3)c4cccc(c4)Cl
22	Mpro-P0157	PET-UNK-29afea89- 2	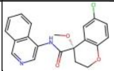	-9.66	-11.27	8/ref*	CO[C@]1(CCOc2c1cc(cc2)Cl)C(=O)Nc3cncc4c3cccc4
23	Mpro-x12000	MAT-POS-199e2e7c- 1	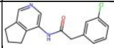	-7.22	-10.63	9	c1cc(cc(c1)Cl)CC(=O)Nc2cncc3c2CCC3

24	Mpro-x11790	PET-UNK-1901c25b-1	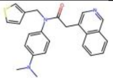	-8.94	-15.09	8	CN(C)c1ccc(cc1)N(Cc2ccsc2)C(=O)Cc3cncc4c3cccc4
25	Mpro-x10201	EDG-MED-0da5ad92- 2	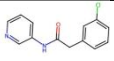	-5.83	-10.59	9	c1cc(cc(c1)Cl)CC(=O)Nc2cccnc2
26	Mpro-x10466	JAN-GHE-5a013bed- 2	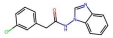	-6.94	-13.89	9	c1ccc2c(c1)ncn2NC(=O)Cc3cccc(c3)Cl
27	Mpro-x10423	JAN-GHE-83b26c96-8	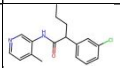	-6.50	-11.86	9	CCCC(c1cccc(c1)Cl)C(=O)Nc2cnccc2C
28	Mpro-x2649	TRY-UNI-714a760b-18	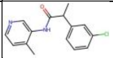	-6.26	-10.49	9	Cc1ccncc1NC(=O)C(C)c2cccc(c2)Cl
29	Mpro-P2243	EDJ-MED-c3ea9889- 6	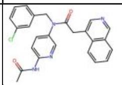	-5.62	NaN[**]	NaN	FC=1C=CC=2C(NC(=O)C3(CC4C3)C=5C=C(Cl)C=CC5O4)=CN=CC2C1
30	Mpro-P2182	MAT-POS-e119ab4f- 2	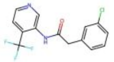	-9.45	NaN	NaN	CNC(=O)CN1CC(C(=O)NC=2C=NC=C3C=CC(F)=CC23)C=4C=C(Cl)C=CC4C1=O
31	Mpro-P2291	EDJ-MED-43f8f7d6-4	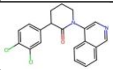	-9.49	NaN	NaN	ClC=1C=CC=2C(=O)N(CC(=O)NC3CC3)CC(C(=O)NC=4C=NC=C5C=CC=CC45)C2C1
32	Mpro-P2402	EDJ-MED-12c4873b-2	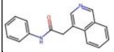	-9.91	NaN	NaN	CNC(=O)C1(CC1)N2CC(C(=O)NC=3C=NC=C4C=CC=CC34)C=5C=C(Cl)C=CC5C2=O

33	Mpro-P1474	MAT-POS-afb6844f-1	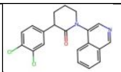	-6.13	NaN	NaN	CNC=1C=NC=C(NC(=O)C2CCOC=3C=CC(Cl)=CC23)C1C
34	Mpro-P0208	EDG-MED-971238d3-4	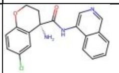	-5.90	-10.14	8	c1ccc2c(c1)cncc2NC(=O)[C@@]3(CCOc4c3cc(cc4)Cl)N
35	Mpro-P0186	ALP-POS-c3a96089- 4	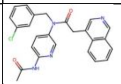	-8.43	-14.06	8	CC(=O)Nc1ccc(cn1)N(Cc2cccc(c2)Cl)C(=O)Cc3cncc4c3cccc4
36	Mpro-P0143	VLA-UCB-34f3ed0c- 11	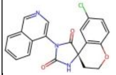	-6.40	-10.89	8	c1ccc2c(c1)cncc2N3C(=O)[C@@]4(CCOc5c4cc(cc5)Cl)NC3=O
37	Mpro-x12207	EDJ-MED-e4b030d8- 13	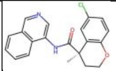	-8.94	-14.84	8	C[C@]1(CCOc2c1cc(cc2)Cl)C(=O)Nc3cncc4c3cccc4
38	Mpro-x11742	MAT-POS-f7918075- 8	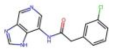	-6.66	-11.46	9	c1cc(cc(c1)Cl)CC(=O)Nc2cncc3c2[nH]cn3
39	Mpro-x11513	ALP-POS-f13221e1- 4	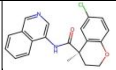	-5.47	NaN	NaN	CC=1C=CN=CC1NC(=O)CCC=2C=CC=C(Cl)C2
40	Mpro-x11271	MAT-POS-c9973a83- 1	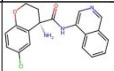	-6.06	NaN	NaN	COC=1C=C(Cl)C=C(CC(=O)NC=2C=NC=CC2C)C1
41	Mpro-P0925	SAM-UNK-2684b532-12	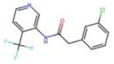	-6.47	-10.88	9	c1cc(cc(c1)Cl)CC(=O)Nc2cnccc2C(F)(F)F

42	Mpro-x11313	EDJ-MED-6af13d92- 1	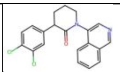	-6.79	NaN	NaN	COC=1C=CC=CC1OCCNC(=O)C2=CC(=O)NC=3C=CC=C(C)C23
43	Mpro-x10355	MAT-POS-590ac91e- 32	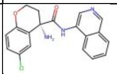	-5.47	NaN	NaN	COC=1C=CC=CC1OCCNC(=O)C2=CC(=O)NC=3C=C(F)C=CC23
44	Mpro-x10723	TRY-UNI-2eddb1ff- 2	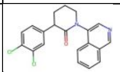	-5.92	NaN	NaN	CC=1C=CN=CC1NC(=O)CC=2C=NC=C(Cl)C2
45	Mpro-x2912	TRY-UNI-714a760b-22	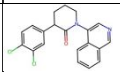	-5.82	NaN	NaN	CC(C(=O)NC=1C=NC=CC1C)C=2C=CC=C(C#N)C2
46	Mpro-x2563	DAR-DIA-23aa0b97- 20	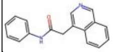	-5.79	-11.45	8	c1ccc(cc1)NC(=O)Cc2cncc3c2cccc3
47	Mpro-P0640	MAT-POS-e9e99895- 2	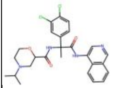	-7.12	-10.52	8	CC(C)N1CCOC(C1)C(=O)NC(C)(c2ccc(c(c2)Cl)Cl)C(=O)Nc3cncc4c3cccc4
48	Mpro-P0145	BRU-CON-c4e3408a- 1	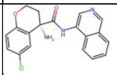	-6.98	NaN	NaN	CC=1C=CN=CC1NC(=O)C2CCOC=3C=CC(Cl)=CC23
49	Mpro-P0950	APL-POS-e0fe77e5- 13	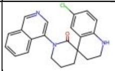	-8.04	-13.14	8	c1ccc2c(c1)cncc2N3CCCC4(C3=O)CCNc5c4cc(cc5)Cl
50	Mpro-x10820	ALP-POS-c59291d4- 4	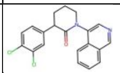	-7.32	NaN	NaN	CNC=1C=CC(=CC1)N(CC=2C=CSC2)C(=O)CN3N=NC=4C=CC=CC34

51	Mpro-P0600	ALP-UNI-3735e77e-1	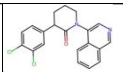	-7.97	-12.59	8	c1ccc2c(c1)cncc2N3CCCC(C3=O)c4ccc(c(c4)Cl)Cl
52	Mpro-P2070	MAT-POS-bfd29aac- 1	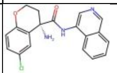	-7.64	NaN	NaN	CN1N=CC=2C=NC=C(NC(=O)C3CN(CC=4C=CC(Cl)=CC34)S(=O) (=O)CC5(CC5)C#N)C12
53	Mpro-x11294	EDJ-MED-6af13d92- 3	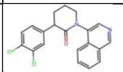	-7.77	NaN	NaN	COC=1C=CC=CC1OCCNC(=O)C2=CC(=O)NC=3C=CC=C(OC)C23
54	Mpro-P0047	LON-WEI-adc59df6- 47	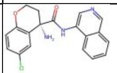	-7.55	NaN	NaN	CCC(=O)N(C(C(=O)NCCC1=CC(F)=CC=C1)C1=CN=CC=C1)C1=CC=C(C=C1)C(C) (C)C
55	Mpro-x10876	ALP-POS-d2866bdf- 1	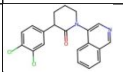	-7.85	NaN	NaN	CN(C)C1=CC=C(C=C1)N(CC1=CSC=C1)C(=O)CN1N=NC2=C1C=CC=C2
56	Mpro-x10959	ADA-UCB-6c2cb422- 1	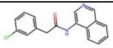	-8.38	-12.67	9/ref*	c1ccc2c(c1)cncc2NC(=O)Cc3cccc(c3)Cl
57	Mpro-P1800	VLA-UCB-50c39ae8- 2	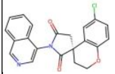	-9.02	-15.22	11A/ref*	c1ccc2c(c1)cncc2N3C(=O)C[C@@]4(C3=O)CCOc5c4cc(cc5)Cl
58	Mpro-P0154	EDG-MED-70ae9412-1	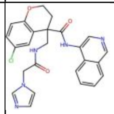	-7.57	-12.30	8	c1ccc2c(c1)cncc2NC(=O)C3(CCOc4c3cc(cc4)Cl)CNC(=O)Cn5ccnc5
59	Mpro-x11642	MAT-POS-6344a35d-1	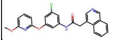	-7.40	-11.64	8	COc1cccc(n1)Oc2cc(cc(c2)Cl)NC(=O)Cc3cncc4c3cccc4

60	Mpro-P0904	EDG-MED-ba1ac7b9- 19	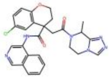	-7.64	-11.30	8	CC1c2nncn2CCN1C(=O)CC3(CCOc4c3cc(cc4)Cl)C(=O)Nc5cncc6c5cccc6
61	Mpro-P0061	RAL-THA-2d450e86-26	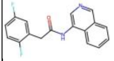	-7.87	-13.20	8	c1ccc2c(c1)cncc2NC(=O)Cc3cc(ccc3F)F
62	Mpro-x11507	ALP-POS-0c2c77e1- 1	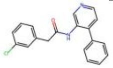	-5.99	-9.60	9	c1ccc(cc1)c2ccncc2NC(=O)Cc3cccc(c3)Cl
63	Mpro-x12073	MAT-POS-8a69d52e- 7	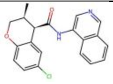	-7.87	-13.94	5	C[C@@H]1COc2ccc(cc2[C@@H]1C(=O)Nc3cncc4c3cccc4)Cl
64	Mpro-x11810	PET-UNK-3c72d439-1	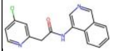	-7.47	-12.31	8	c1ccc2c(c1)cncc2NC(=O)Cc3cc(ccn3)Cl
65	Mpro-P0831	MAT-POS-96f51285-5	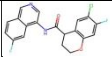	-8.88	-14.33	8	c1cc2cncc(c2cc1F)NC(=O)C3CCOc4c3cc(c(c4)F)Cl
66	Mpro-P0816	EDG-MED-5d232de5- 8	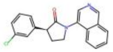	-6.01	-10.48	8	c1ccc2c(c1)cncc2N3CC[C@H](C3=O)c4cccc(c4)Cl
67	Mpro-x12695	MAT-POS-c7771779- 1	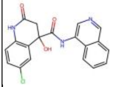	-6.39	-10.03	8	c1ccc2c(c1)cncc2NC(=O)C3(CC(=O)Nc4c3cc(cc4)Cl)O
68	Mpro-P0845	ALP-POS-9c80c481-1	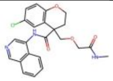	-7.84	-12.17	8	CNC(=O)COCC1(CCOc2c1cc(cc2)Cl)C(=O)Nc3cncc4c3cccc4

69	Mpro-x11789	MAT-POS-bfb445d4- 2	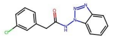	-7.15	-15.37	9	c1ccc2c(c1)nnn2NC(=O)Cc3cccc(c3)Cl
70	Mpro-P1079	MAT-POS-4223bc15-40	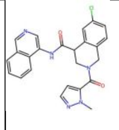	-9.30	-16.20	8	Cn1c(ccn1)C(=O)N2Cc3ccc(cc3C(C2)C(=O)Nc4cncc5c4cccc5)Cl
71	Mpro-x11508	EDJ-MED-50fe53e8- 1	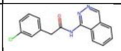	-6.37	-13.38	9	c1ccc2c(c1)cnnc2NC(=O)Cc3cccc(c3)Cl
72	Mpro-P0243	EDJ-MED-d08626de- 3	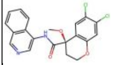	-8.89	-14.80	8	CO[C@@]1(CCOc2c1cc(c(c2)Cl)Cl)C(=O)Nc3cncc4c3cccc4
73	Mpro-P0188	MAT-POS-5d65ec79- 2	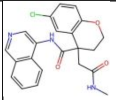	-7.48	-11.74	8	CNC(=O)CC1(CCOc2c1cc(cc2)Cl)C(=O)Nc3cncc4c3cccc4
74	Mpro-x11548	MIC-UNK-08cd9c58- 1	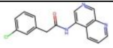	-5.70	-9.36	9	c1cc(cc(c1)Cl)CC(=O)Nc2cncc3c2cccn3
75	Mpro-x11498	VLA-UCB-1dbca3b4- 15	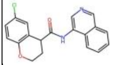	-8.80	-15.08	5	c1ccc2c(c1)cncc2NC(=O)C3CCOc4c3cc(cc4)Cl
76	Mpro-x11488	MAT-POS-1e5f28a7- 1	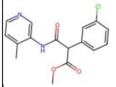	-6.32	-11.01	9	Cc1ccncc1NC(=O)C(c2cccc(c2)Cl)C(=O)OC
77	Mpro-P0068	RAL-THA-2d450e86-17	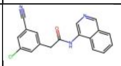	-8.69	-14.41	8	c1ccc2c(c1)cncc2NC(=O)Cc3cc(cc(c3)Cl)C#N

78	Mpro-P0124	BEN-DND-c852c98b-5	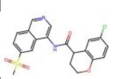	-8.21	-13.07	8	CS(=O)(=O)c1ccc2cncc(c2c1)NC(=O)C3CCOc4c3cc(cc4)Cl
79	Mpro-x10422	JAN-GHE-83b26c96-22	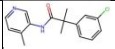	-5.48	-9.78	9	Cc1ccncc1NC(=O)C(C)(C)c2cccc(c2)Cl
80	Mpro-x11542	MAT-POS-bb423b95- 1	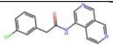	-6.96	-13.73	9	c1cc(cc(c1)Cl)CC(=O)Nc2cncc3c2ccnc3
81	Mpro-x12171	ALP-POS-477dc5b7- 2	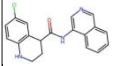	-8.99	-14.35	8	c1ccc2c(c1)cncc2NC(=O)C3CCNc4c3cc(cc4)Cl
82	Mpro-P0185	MAT-POS-fce787c2-6	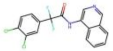	-7.96	-13.43	8	c1ccc2c(c1)cncc2NC(=O)C(c3ccc(c(c3)Cl)Cl)(F)F
83	Mpro-P0811	ALP-POS-477dc5b7- 5	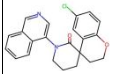	-8.62	-13.19	8	c1ccc2c(c1)cncc2N3CCCC4(C3=O)CCOc5c4cc(cc5)Cl
84	Mpro-x11609	MAT-POS-3b92565d-1	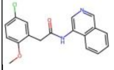	-8.08	-13.78	8	COc1ccc(cc1CC(=O)Nc2cncc3c2cccc3)Cl
85	Mpro-x12692	EDJ-MED-92e193ae- 1	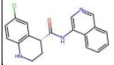	-9.06	-14.76	8	c1ccc2c(c1)cncc2NC(=O)[C@@H]3CCNc4c3cc(cc4)Cl
86	Mpro-x12699	EDG-MED-0e5afe9d- 3	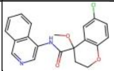	-9.19	-14.74	8	COC1(CCOc2c1cc(cc2)Cl)C(=O)Nc3cncc4c3cccc4

87	Mpro-x11543	ALP-POS-95b75b4d-5	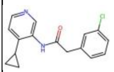	-7.02	-11.10	9	c1cc(cc(c1)Cl)CC(=O)Nc2cnccc2C3CC3
88	Mpro-P0108	RAL-THA-2d450e86-6	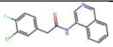	-8.78	-14.45	8	c1ccc2c(c1)cncc2NC(=O)Cc3ccc(c(c3)Cl)F
89	Mpro-P0141	MAT-POS-f9802937- 7	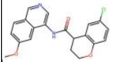	-9.07	-14.58	8	COc1ccc2cncc(c2c1)NC(=O)C3CCOc4c3cc(cc4)Cl
90	Mpro-P0601	EDJ-MED-e4b030d8- 11	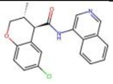	-9.25	-16.79	5	C[C@H]1COc2ccc(cc2[C@@H]1C(=O)Nc3cncc4c3cccc4)Cl
91	Mpro-x11473	PET-UNK-8df914d1- 4	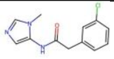	-5.50	-9.95	9	Cn1cncc1NC(=O)Cc2cccc(c2)Cl
92	Mpro-P0996	MAT-POS-4223bc15-30	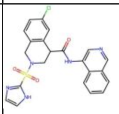	-9.10	-13.32	8	c1ccc2c(c1)cncc2NC(=O)C3CN(Cc4c3cc(cc4)Cl)S(=O)(=O)c5[nH]ccn5
93	Mpro-P0808	MAT-POS-dd3ad2b5- 2	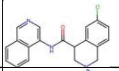	-8.76	-14.10	8	CC(=O)N1Cc2ccc(cc2C(C1)C(=O)Nc3cncc4c3cccc4)Cl
94	Mpro-x12677	MAT-POS-afd4d4fd- 2	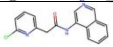	-6.00	-10.61	8	c1ccc2c(c1)cncc2NC(=O)Cc3cccc(n3)Cl
95	Mpro-P0022	VLA-UCB-29506327-1	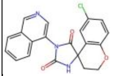	-8.03	-16.04	11A	c1ccc2c(c1)cncc2N3C(=O)C4(CCOc5c4cc(cc5)Cl)NC3=O

96	Mpro-x2646	TRY-UNI-714a760b-6	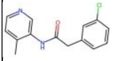	-6.29	-10.84	9	Cc1ccncc1NC(=O)Cc2cccc(c2)Cl
97	Mpro-P0114	ALP-POS-869ac754-1	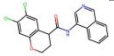	-9.13	-14.47	8	c1ccc2c(c1)cncc2NC(=O)C3CCOc4c3cc(c(c4)Cl)Cl
98	Mpro-x11501	MAT-POS-bb423b95- 7	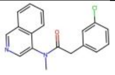	-6.10	-10.84	9	CN(c1cncc2c1cccc2)C(=O)Cc3cccc(c3)Cl
99	Mpro-x11530	MAT-POS-bb423b95- 2	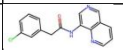	-7.04	-12.17	9	c1cc(cc(c1)Cl)CC(=O)Nc2cncc3c2nccc3
100	Mpro-x11317	TRY-UNI-714a760b-3	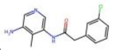	-7.34	-12.51	9	Cc1c(cncc1NC(=O)Cc2cccc(c2)Cl)N
101	Mpro-P0765	BEN-BAS-c2bc0d80- 7	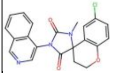	-8.53	-16.92	11A	CN1C(=O)N(C(=O)C12CCOc3c2cc(cc3)Cl)c4cncc5c4cccc5
102	Mpro-x12777	EDJ-MED-00c1612e-1	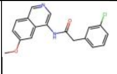	-8.26	-11.71	9	COc1ccc2cncc(c2c1)NC(=O)Cc3cccc(c3)Cl
103	Mpro-P0066	RAL-THA-2d450e86-1	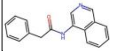	-6.63	-11.17	8	c1ccc(cc1)CC(=O)Nc2cncc3c2cccc3
104	Mpro-P0065	RAL-THA-2d450e86-13	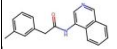	-7.26	-12.13	8	Cc1cccc(c1)CC(=O)Nc2cncc3c2cccc3

105	Mpro-P0148	EDG-MED-5d232de5- 3	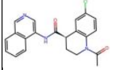	-6.51	-11.02	8	CC(=O)N1CC[C@H](c2c1ccc(c2)Cl)C(=O)Nc3cncc4c3cccc4
106	Mpro-P0160	EDG-MED-5d232de5- 1	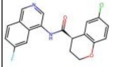	-8.86	-14.93	8	c1cc2cncc(c2cc1F)NC(=O)[C@@H]3CCOc4c3cc(cc4)Cl
107	Mpro-P0130	RAL-THA-05e671eb- 10	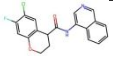	-9.20	-14.62	8	c1ccc2c(c1)cncc2NC(=O)C3CCOc4c3cc(c(c4)F)Cl
108	Mpro-x11499	MAT-POS-f7918075- 5	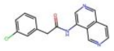	-7.33	-12.60	9	c1cc(cc(c1)Cl)CC(=O)Nc2cncc3c2cncc3
109	Mpro-P0887	RUB-POS-1325a9ea- 4	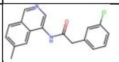	-8.56	-11.88	9	Cc1ccc2cncc(c2c1)NC(=O)Cc3cccc(c3)Cl
110	Mpro-P0777	PET-UNK-bb7ffe78- 1	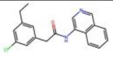	-7.84	-12.98	8	CCc1cc(cc(c1)Cl)CC(=O)Nc2cncc3c2cccc3
111	Mpro-P1470	RUB-POS-1325a9ea- 14	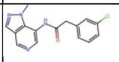	-7.95	-14.25	9	Cn1c2c(cncc2NC(=O)Cc3cccc(c3)Cl)cn1
112	Mpro-x11813	MAR-UCB-f313ec4d- 2	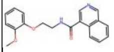	-7.50	-12.87	8	COc1ccccc1OCCNC(=O)c2cncc3c2cccc3
113	Mpro-P1200	MAT-POS-e6dd326d- 6	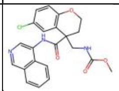	-8.52	-13.30	8	COC(=O)NCC1(CCOc2c1cc(cc2)Cl)C(=O)Nc3cncc4c3cccc4

114	Mpro-P0064	RAL-THA-2d450e86-10	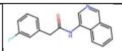	-7.83	-12.92	8	c1ccc2c(c1)cncc2NC(=O)Cc3cccc(c3)F
115	Mpro-P0627	MAT-POS-78e1d523-1	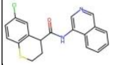	-9.13	-14.14	8	c1ccc2c(c1)cncc2NC(=O)C3CCSc4c3cc(cc4)Cl
116	Mpro-P0121	MAT-POS-fce787c2-3	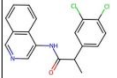	-8.36	-13.14	8	CC(c1ccc(c(c1)Cl)Cl)C(=O)Nc2cncc3c2cccc3
117	Mpro-P0111	ALP-UNI-3735e77e-2	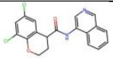	-8.98	-14.10	8	c1ccc2c(c1)cncc2NC(=O)C3CCOc4c3cc(cc4Cl)Cl
118	Mpro-P0069	RAL-THA-2d450e86-7	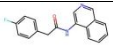	-7.24	-12.16	8	c1ccc2c(c1)cncc2NC(=O)Cc3ccc(cc3)F
119	Mpro-P0872	ALP-UNI-8d415491-6	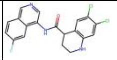	-9.02	-13.32	8	c1cc2cncc(c2cc1F)NC(=O)C3CCNc4c3cc(c(c4)Cl)Cl
120	Mpro-P0607	MAT-POS-a13804f0- 3	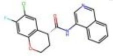	-9.57	-16.48	8	c1ccc2c(c1)cncc2NC(=O)[C@@H]3CCOc4c3cc(c(c4)F)Cl
121	Mpro-P0630	MAT-POS-e9e99895- 11	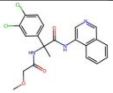	-6.90	-11.07	8	CC(c1ccc(c(c1)Cl)Cl)(C(=O)Nc2cncc3c2cccc3)NC(=O)COC
122	Mpro-P0010	PET-UNK-c9c1e0d8- 4	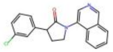	-8.05	-13.10	8	c1ccc2c(c1)cncc2N3CCC(C3=O)c4cccc(c4)Cl

123	Mpro-x12587	PET-UNK-c9c1e0d8- 3	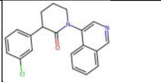	-8.11	-13.11	8	c1ccc2c(c1)cncc2N3CCCC(C3=O)c4cccc(c4)Cl
124	Mpro-P0793	EDG-MED-5d232de5-7	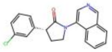	-7.55	-12.29	8	c1ccc2c(c1)cncc2N3CC[C@@H](C3=O)c4cccc(c4)Cl
125	Mpro-x11458	PET-UNK-c9c1e0d8- 2	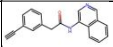	-6.87	-11.47	8	C#Cc1cccc(c1)CC(=O)Nc2cncc3c2cccc3
126	Mpro-x11812	MAR-UCB-f313ec4d- 6	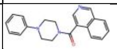	-7.19	-12.23	8	c1ccc(cc1)N2CCN(CC2)C(=O)c3cncc4c3cccc4
127	Mpro-P0578	RAL-THA-4aa06b95- 7	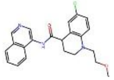	-8.53	-13.12	8	COCCN1CCC(c2c1ccc(c2)Cl)C(=O)Nc3cncc4c3cccc4
128	Mpro-P1477	MAT-POS-a13804f0- 4	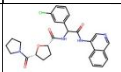	-8.54	-14.41	8	c1ccc2c(c1)cncc2NC(=O)C(c3cccc(c3)Cl)NC(=O)[C@@H]4CC[C@@H] (O4)C(=O)N5CCCC5
129	Mpro-P0126	RAL-THA-2d450e86-30	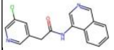	-8.19	-13.52	8	c1ccc2c(c1)cncc2NC(=O)Cc3cc(cnc3)Cl
130	Mpro-P0135	MAT-POS-de59a476- 2	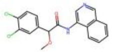	-8.15	-13.05	8	COC(c1ccc(c(c1)Cl)Cl)C(=O)Nc2cncc3c2cccc3

B Supporting Results

B.1 Density-based clustering yields similar kinetics as K-means clustering with fewer microstates

With APLoD clustering, implied timescales plateau after 20 to 30 steps, or 2 to 3 ns (***Figure S1***). Thus, we used a lag time of 3 ns, shorter than the 4 ns lag time we used in previous work based on K-means clustering.[35] MSMs that maximize the variational approach for Markov processes-2 (VAMP-2) score[52] have low discretization error. With only 91 microstates, the MSM built with the density-based clustering method APLoD yielded a VAMP-2 score of 9.84. In contrast, a MSM built with K-means clustering with 100 microstates produced a slightly lower VAMP-2 score of 9.74. These values are comparable to the VAMP-2 score of 9.92 that we previously reported for a MSM based on K-means clustering with 2500 microstates,[35] indicating that the new MSM provides a good representation of the system kinetics. The Chapman–Kolmogorov test at a lag time of 3 ns (***Figure S2***) shows close agreement between estimated and predicted transition probabilities, validating that the MSM accurately reproduces the original system dynamics.

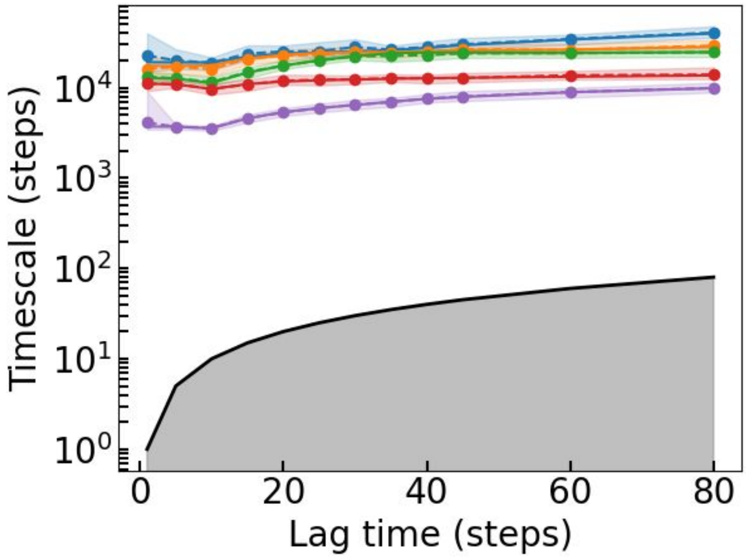

Figure S1. Implied timescale analysis used to select the MSM lag time. The first five slowest relaxation processes are shown as colored curves, plotted as a function of lag time (in steps; 1 step = 0.1 ns) on a logarithmic scale. The shaded gray region indicates lag times below the reliably Markovian regime. Each curve represents a slow process without implying a specific conformational assignment.

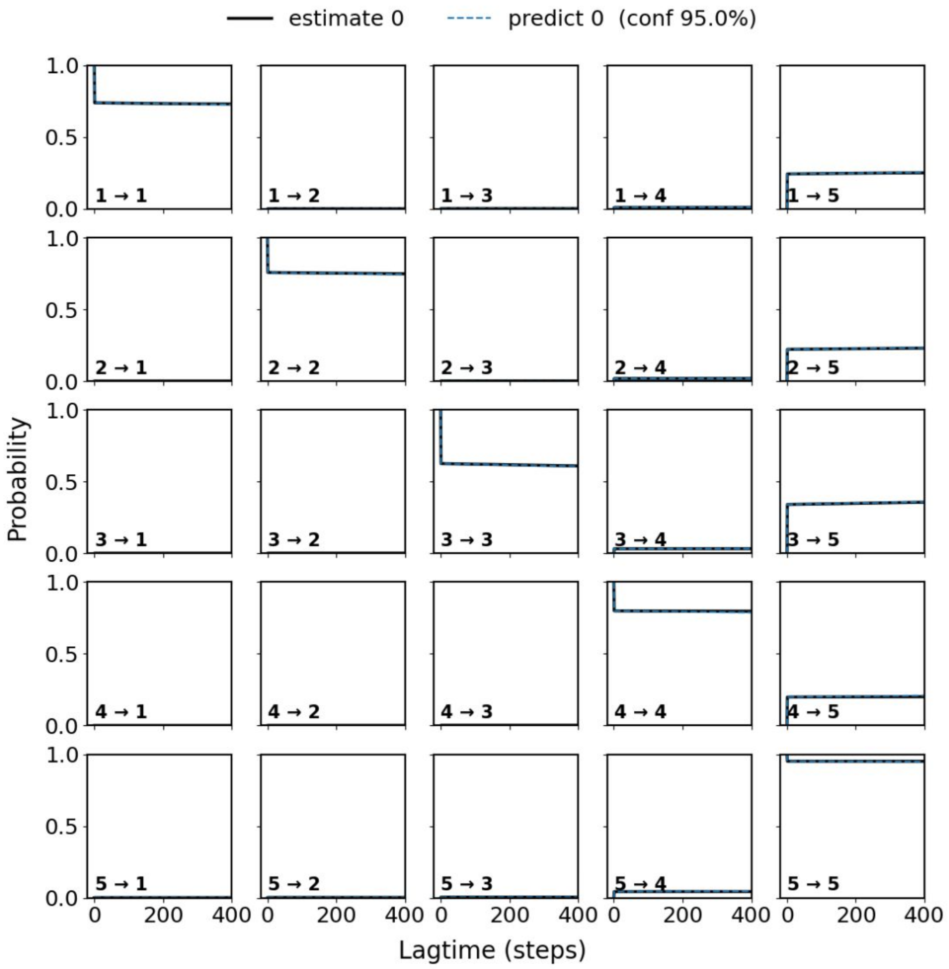

Figure S2. Chapman–Kolmogorov (CK) test for the five-state Markov state model at a lag time of 30 steps (3 ns). PCCA+ was used to do coarse-graining for lumping 91 microstates into five macrostates. The number of states was chosen based on the convergence of the slowest implied timescales. Solid black lines denote the directly estimated transition probabilities, while blue dashed lines indicate the MSM predictions with 95% confidence intervals. Each subplot represents the transition probability from state that the built MSM reproduces the dynamics of the original system.

B.2 The computational cost of AlGDock is much more than molecular docking

AlGDock is more computationally intensive than conventional docking methods such as DOCK6 and Vina. BPMF calculations require 10-24 hours of CPU time on a single core. In contrast, Vina completed 130 ligand–snapshot docking jobs in 104.98 minutes using 8 CPU cores on an AMD Opteron 6376 processor, corresponding to 0.107 CPU-core hours per receptor-ligand pair. DOCK6 completed 130 jobs on the same systems with an average of 5.34 minutes per ligand on a single core of an AMD EPYC Milan processor, or 0.089 CPU-core hours per job. Thus, AlGDock requires about two orders of magnitude more compute time.

B.3 BPMF calculations are converged

The reproducibility of the absolute binding free energy calculations was validated by monitoring BPMF estimates in 40 of 130 systems. For each system, five independent replicas were executed, and the convergence of the energy values was tracked through 20 cycles of the CD thermodynamic process (interaction grid scaling). On average, the BPMF estimations stabilized to within 1 kcal/mol within 20 cycles, with the ensemble-wide convergence fraction reaching approximately 80% (***Figure S3***).

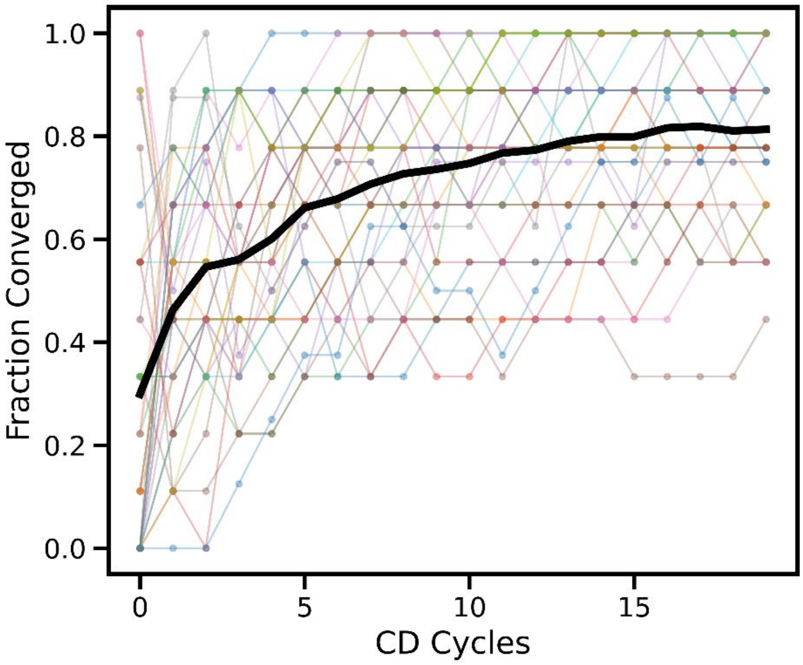

Figure S3. Ensemble convergence behavior during interaction grid scaling (CD). The plot displays the fraction of converged BPMF calculations over 20 CD cycles. Each transparent colored line represents the convergence fraction of an individual system, calculated as the mean of its five independent replicas. The bold black line denotes the grand mean across all 40 systems.

B.4 The accuracy of ΔG calculations depended on the threshold energy and snapshot selection scheme, but not MSM weights

We evaluated the impact of the energy threshold (δ) and snapshot selection scheme on correlation metrics and errors. For all snapshot selection schemes, the accuracy of

ΔG calculations generally increased with the energy threshold until converging to an asymptote (***Figure S4***). Increasing the energy threshold increases the number of BPMFs included in the ΔG calculation. For the Docking and Random Crystal schemes, performance converged at around δ = 10 kcal/mol, above which a higher threshold provided minimal benefits. For the Full Crystal scheme, most metrics converged with δ from 15 kcal/mol, but ρ was still higher at δ = 20 kcal/mol. As the performance of the Random Crystal scheme depends on the specific set of selected structures, this scheme showed a larger variability in performance that diminishes as the threshold (and the number of structures) increases.

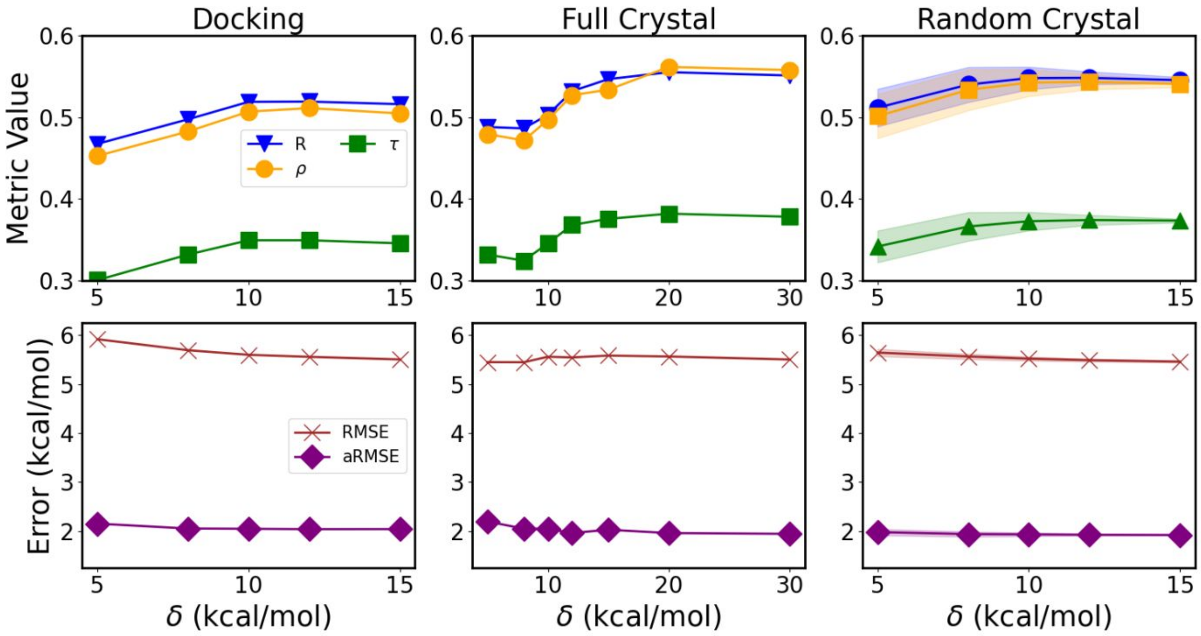

Figure S4. Performance of ΔG calculations across different snapshot selection schemes as a function of threshold (δ). Calculations were done for the 57 small molecules from the first round of ligand preparation. The top row of plots shows correlation metrics (Pearson R, Spearman ρ, and Kendall τ) for Docking, Full Crystal, and Random Crystal schemes. The bottom row shows error metrics (RMSE and aRMSE). In the Random Crystal scheme, the shaded regions represent 95% confidence intervals from bootstrapping. Bootstrapping was performed using different 1000 sets of 12 randomly selected ligands.

Compared to the other schemes, the Full Crystal scheme required a larger energy threshold δ for convergence because of differences in the minimization procedure. The default behavior of DOCK6, which is used in the Docking scheme, is to minimize the position of anchor moieties but not the entire molecule.[1] In contrast, the Full Crystal scheme is based on an optimization of the entire ligand, allowing it to make more subtle adjustments to reduce its interaction energy with specific receptor snapshots. Thus, a higher energy threshold δ was required to include all the receptor conformations relevant for convergence, despite still using fewer snapshots than the Docking or Random Crystal schemes (***Figure S5***). While the Random Crystal scheme also includes optimization of entire ligands, receptor snapshots were selected based

on several structures with different ligands, providing multiple opportunities to select receptor snapshots relevant for convergence.

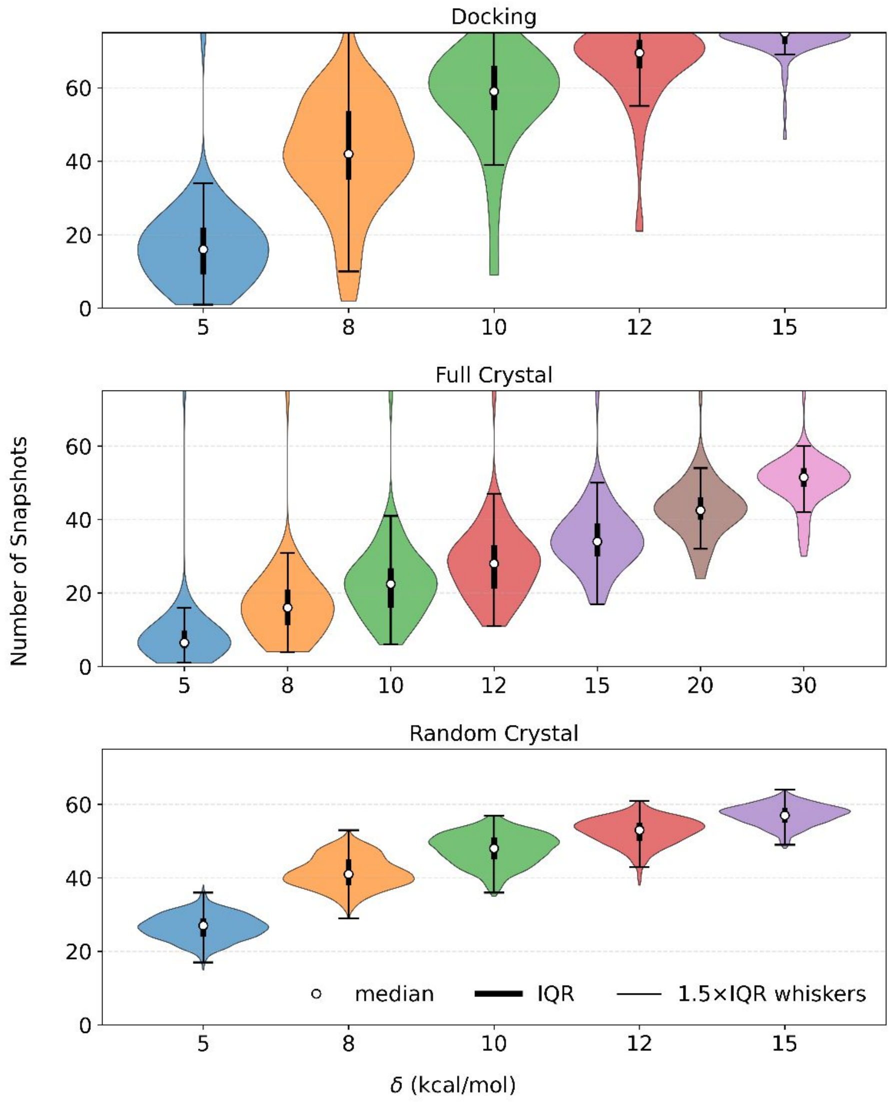

Figure S5. Distributions of selected snapshot counts across energy thresholds (δ) for the three schemes. Each violin shows the kernel-density of counts at a given threshold; the white dot marks the median, the thick black segment spans the interquartile range (IQR), and thin black whiskers extend to 1.5×IQR.

Once convergence was achieved, the Full Crystal scheme was most accurate, followed by Random Crystal and then by Docking. Trends for the initial set of 57 ligands (***Figure S4***) were also observed for the complete set of 130 ligands. Scatter plots comparing calculated and experimental ΔG for the complete set were shown for the Full Crystal scheme at δ = 15 kcal/mol (***Figure 2a***) and Random Crystal and Docking schemes at δ = 10 kcal/mol (***Figure S6***). At these thresholds, the Full Crystal scheme produced calculations with higher correlation (R=0.55, ρ=0.54, τ=0.37) than the Random Crystal (R=0.5, ρ=0.48, τ=0.33) and Docking (R=0.35, ρ=0.39, τ=0.25) schemes. The Random Crystal scheme required approximately 12 crystal structures to achieve moderate correlation, beyond which additional structures provided minimal improvement (***Figure S7***).

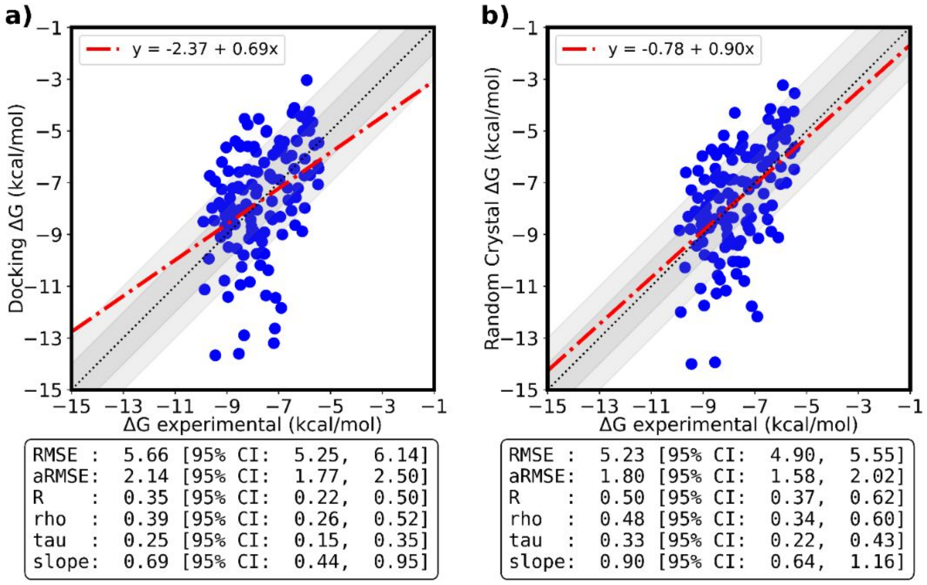

Figure S6. Alchemical free-energy predictions using a) Docking and b) Random Crystal scheme (AlGDock). The δ=10 kcal/mol was used as threshold for snapshot selection, the estimations were for all 130 small molecules. The predictions have been shifted by their average offset from experiment. Statistical performance with 95% confidence intervals (CI) is shown in the table below the plot. The dark gray and light gray bands indicate ±1 kcal/mol and ±2 kcal/mol deviations from the regression line, respectively, while the red dashed line represents the best-fit linear regression to the data.

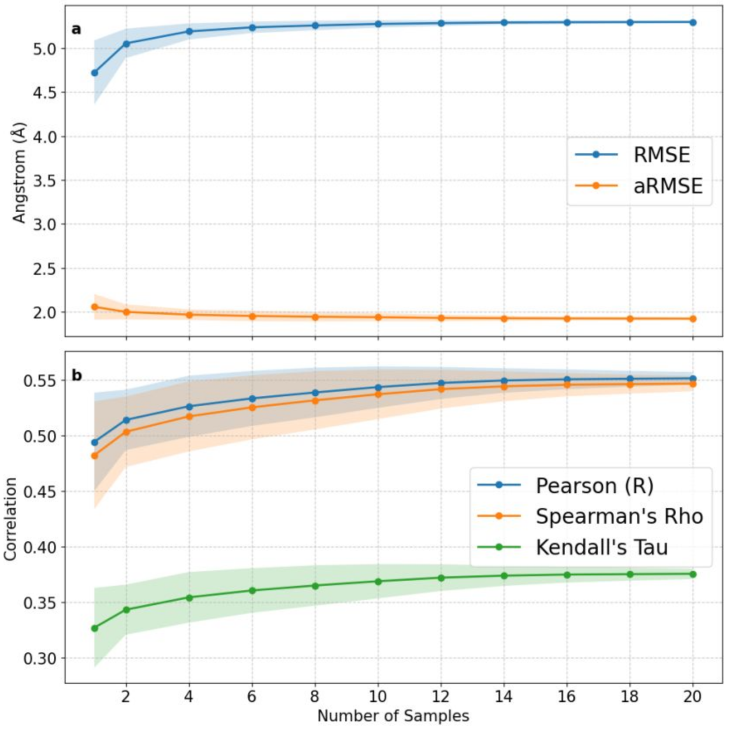

Figure S7. Correlation metrics as a function of the number of crystal structures. The mean (line and markers) and standard deviation (shaded regions) of (a) RMSE, aRMSE, (b) Pearson R, Spearman ρ, and Kendall τ as function of sample size. The energy threshold was set at δ = 10 kcal/mol. Standard deviations were based on bootstrap replicates.

Converted ΔG calculations preserve the performance ranking observed in their counterparts based on direct ΔG calculations (***Figure S8***). As we observed with Full

Crystal (***Error! Reference source not found.b***), *Converted* results showed improved correlation metrics (increased by ~0.05 to 0.1) for all three schemes, demonstrating enhanced ability to rank binding affinities. However, this improvement came at the cost of increased aRMSE, indicating a loss in accuracy in ΔG.

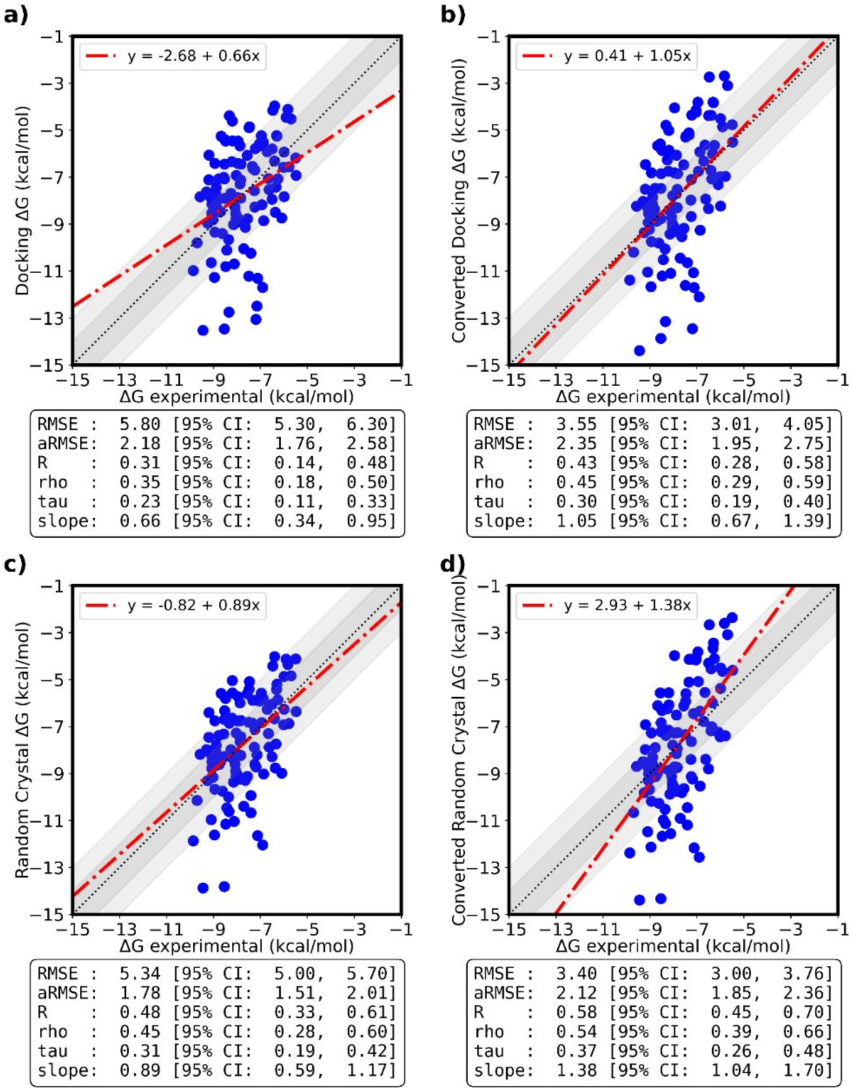

Figure S8. Alchemical free-energy predictions using (a) Docking, (b) Converted Docking, (c) Random Crystal and (d) Converted Random Crystal. *All schemes were applied to the set of 110 small molecules with available FEP data. As in Figure S2, all predictions have been shifted by their average offset from the experiments. Statistical performance with 95% confidence intervals (CI) appears in the table below each plot. The dark gray and light gray bands represent ±1 kcal/mol and ±2 kcal/mol deviations from the regression line, respectively, while the red dashed line shows the best-fit linear regression for each panel.*

We were curious about the importance of MSM-derived weights to the accuracy of our ΔG calculations. In ensemble docking, the method used to aggregate scores across the ensemble is a critical decision. Common approaches include averaging docking scores, selecting the minimum score,[2,3] or computing an exponential average of BPMFs, as in ILT.[4] According to ILT (Eq. 4), the estimated binding free energy is an exponential average over receptor snapshots in the apo ensemble. To evaluate the influence of this weight on the accuracy of ΔG calculations, we compared calculations

based on MSM-derived equilibrium probabilities or equal weights for all receptor snapshots. Performance outcomes of both approaches were nearly identical (***Table S2***). The correlation coefficients for binding free energy predictions across different snapshot selection schemes remained virtually unchanged, regardless of whether MSM weights were used or not. This was attributed to the near-uniform distribution of weights across fingerprint-based clusters, which averaged out distinctions between high- and low-probability MSM states (***Figure S9***).

The negligible influence of receptor snapshot weights was due to minimal variation in the equilibrium probabilities of binding pocket conformations. While MSM microstate probabilities were highly non-uniform (***Figure S9a***), distributing these probabilities across snapshots in each microstate led to nearly uniform weights of clusters based on occupancy fingerprints (***Figure S9b***). This occurred because each fingerprint-based cluster included snapshots from both high- and low-probability MSM states, effectively averaging out their distinctions. Consequently, although the MSM captures meaningful thermodynamic and kinetic information, its influence was diminished, resulting in similar binding free energy estimates regardless of whether MSM weights were applied or not (***Table S2***).

Table S2. Effects of MSM weights on exponential averages of BPMFs. Comparison of correlation coefficients and error metrics between two approaches for calculating exponential averages of BPMFs: using MSM-derived weights versus equally weighting snapshots, across different snapshot filtering schemes. Metrics include Pearson correlation coefficient (R), root mean square error (RMSE), adjusted RMSE (aRMSE), Spearman's rank correlation coefficient (ρ), and Kendall's tau (τ).

	Snapshot filtering	R	RMSE	aRMSE	ρ (rho)	τ (tau)
MSM-weighted snapshots	DOCKING	0.36	5.72	2.11	0.40	0.26
	FULL Crystals	0.56	5.03	1.58	0.54	0.38
	RANDOM Crystals	0.50	5.27	1.78	0.48	0.33
Equally weighted snapshots	DOCKING	0.35	5.66	2.14	0.39	0.25
	FULL Crystals	0.57	4.95	1.58	0.54	0.38
	RANDOM Crystals	0.50	5.23	1.80	0.48	0.33

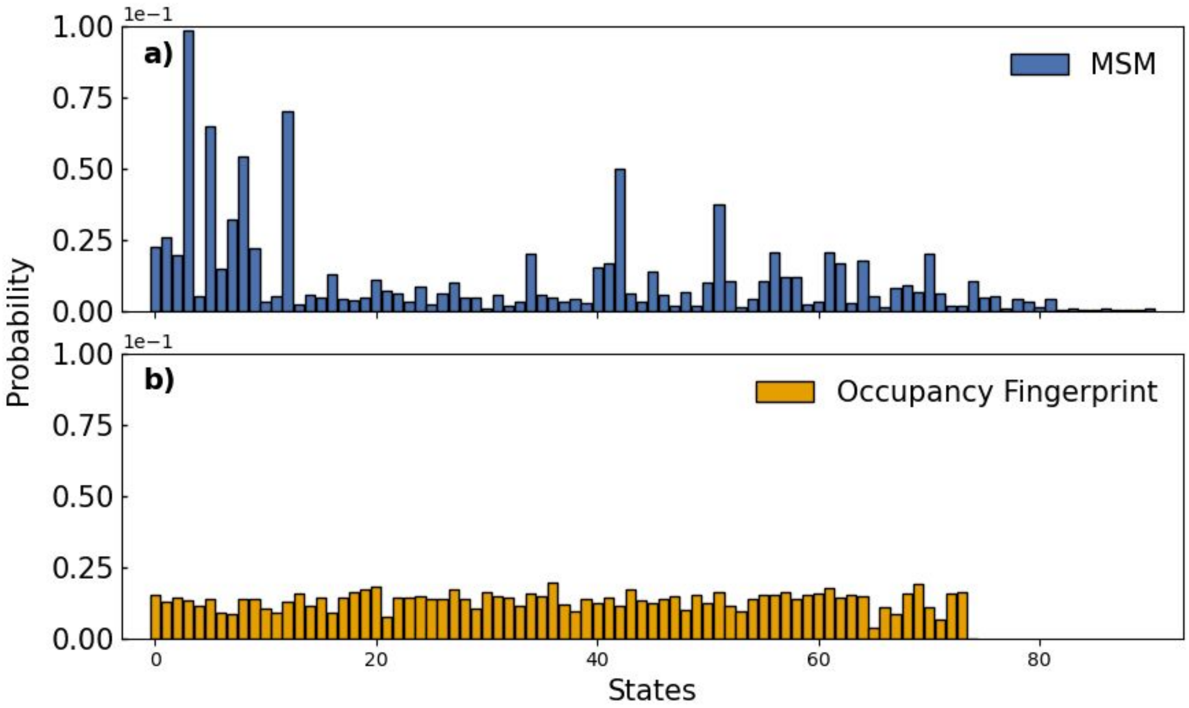

Figure S9. Histograms of weights for receptor states. (a) Stationary distribution of MSM microstates derived from whole-system torsion angles. (b) Reweighted clusters based on binding-site occupancy fingerprints. State indices are arbitrary.

B.5 AIGDock recapitulates major structure-activity relationships

While a comprehensive analysis is outside the scope of this work, we selected four lead compounds over a broad range of affinity to evaluate structure-activity relationship (SAR) trends. Generally, the COVID Moonshot compounds share a common pharmacophore: a H-bond acceptor, such as a pyrazole moiety, forms a critical hydrogen bond with the Nϵ2 of H163, while the linker ketone is stabilized by an H-bond to the backbone nitrogen of E166. Furthermore, two aromatic moieties flanking the linker are stabilized by π-stacking with H41 or non-polar interactions with E166 and adjacent residues. Of these four, the weakest binder, Mpro-x11473 (-5.5 kcal/mol), demonstrates the baseline requirements for binding but lacks peripheral stabilization (***Figure S10a)***. Its non-polar groups are left exposed to the large, solvent-accessible volume of the MPro pocket, resulting in a weak overall affinity. For the moderate binder, Mpro-P0022 (-8.02 kcal/mol), higher potency may be attributed to expansion of the aromatic scaffold, which enhances hydrophobic packing against the E166 sidechain and adjacent residues, alongside the recruitment of new hydrogen bonds with C145, or structural water molecules (***Figure S10b***). The most potent representative leads, Mpro-P2263 (-9.45 kcal/mol) and Mpro-P2017 (-9.86 kcal/mol) introduce an electrophilic sulfonyl group and rigid hydrophobic motifs, specifically the cyclopropyl and cyclobutyl rings. These motifs not only expand the hydrophobic contact surface with MPro but also facilitate a complex H-bond network with the solvent and adjacent residues. Interestingly, a subtle difference of approximately -0.4 kcal/mol exists between (c) and (d). This small gap likely arises from the slightly more rigid architecture of (c), which incorporates a pyrrolidinone linker (***Figure S10c***). This increased rigidity potentially restricts the ligand's ability to optimize its solvent-mediated interactions compared to the slightly more adaptable coordination observed in (d). Specifically, the flexibility of (d) enables it to recruit a structural water molecule that bridges a critical interaction with the Q189 side chain (***Figure S10d***).

This SAR logic is remarkably consistent with the ΔG values obtained via AIGDock calculations, which correctly ranked Mpro-x11473 (a) as the weakest (-8.70 kcal/mol), Mpro-P0022 (b) as moderate (-11.47 kcal/mol), and Mpro-P2263 (c), Mpro-P2017 (d) as the strongest binders (-18.25 and -18.05 kcal/mol, respectively). While AIGDock systematically overestimates binding magnitudes, it successfully reproduces the relative ranking of the diverse scaffolds. Interestingly, the small experimental difference of -0.4 kcal/mol between (c) and (d) is mirrored by a negligible -0.2 kcal/mol gap in the computational model. Although AIGDock slightly favors the more rigid architecture of (c), experimental data suggests that the adaptability of (d) may allow for more optimal solvent-mediated interactions. Despite the inherent limitations of the implicit solvent model in capturing fine-grained solvent dynamics, these results demonstrate AIGDock’s ability to identify core interactions and rank small-molecule inhibitors.

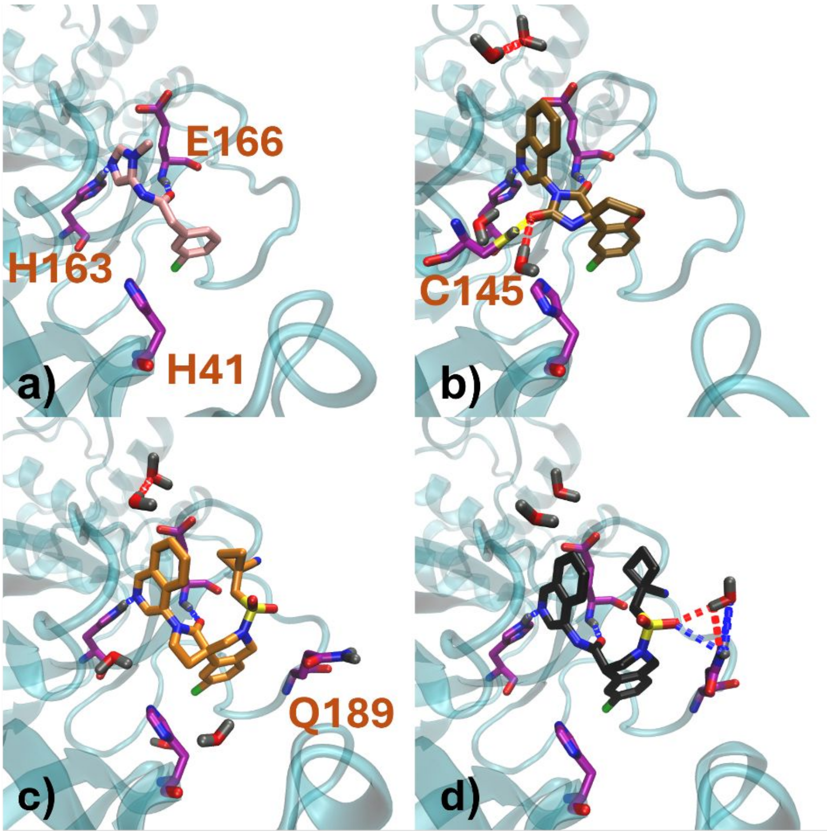

*Figure S10. Structure-activity relationship (SAR) analysis of representative MPro inhibitors. (a–d) Crystal structures of four lead compounds: **(a)** Mpro-x11473 (PDB entry 7GEK), **(b)** Mpro-P0022 (PDB entry GHU), **(c)** Mpro-P2263 (PDB entry 7GN1), and **(d)** Mpro-P2017 (PDB entry 7GLT). Compounds are ordered by increasing binding affinity (∆G), ranging from the weakest **(a)** to the strongest **(d)**. The MPro protein is shown in cyan, with key residues H163 and E166 highlighted with purple carbons. Ligands are colored pink **(a)**, brown **(b)**, orange **(c)**, and black **(d)**. Standard atom coloring is used for other elements: H (grey), O (red), N (blue), Cl (green), and S (yellow). Hydrogen bonds are colored based on the donor atom's color.*

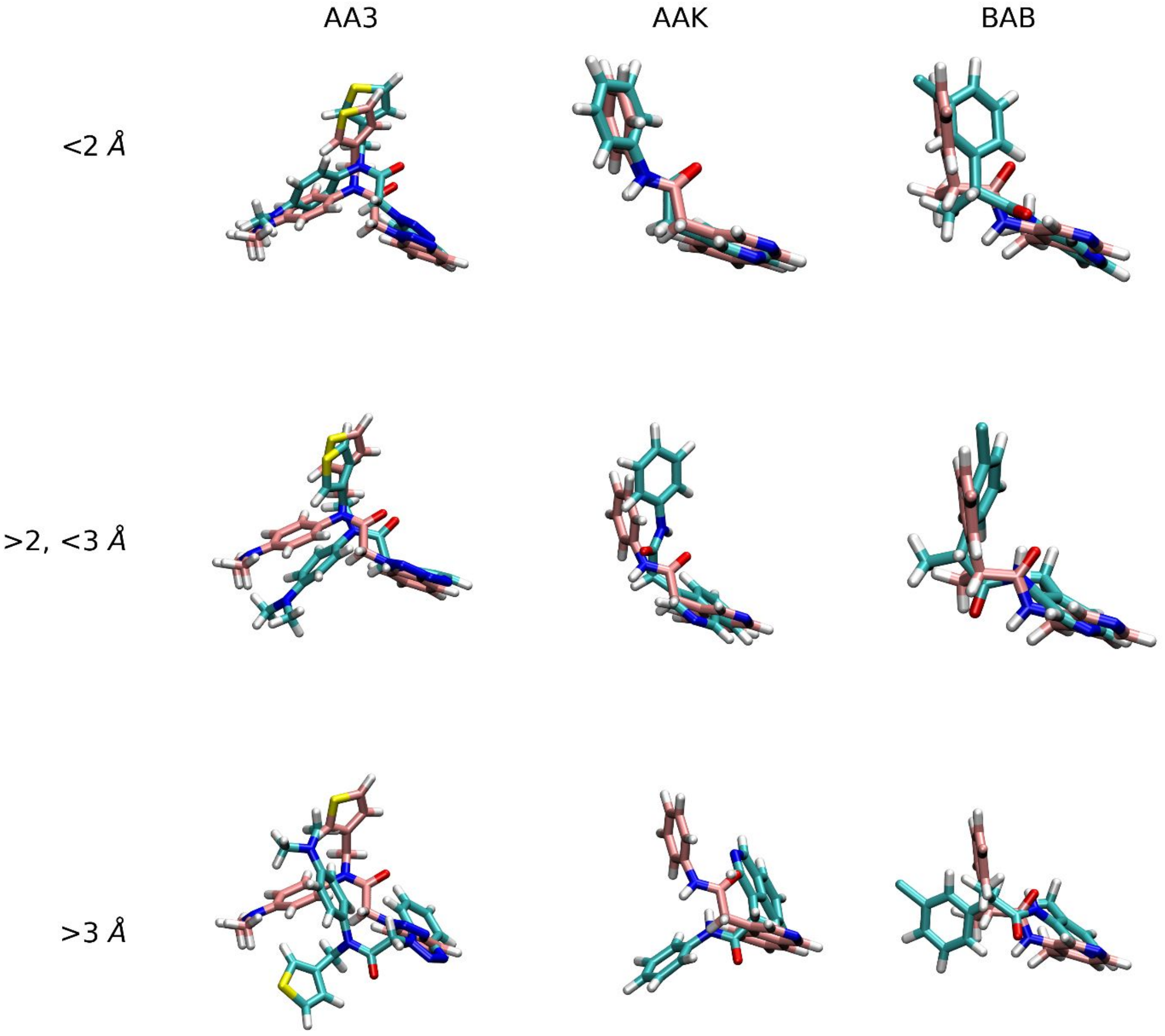

Figure S11. Comparison of predicted and crystallographic ligand poses across different RMSD ranges. The comparison used three representative compounds (AA3, AAK, and BAB). Each panel shows the superposition of the predicted pose (cyan) and the crystallographic pose (pink). Poses are grouped by RMSD: <2 Å (top row), between 2–3 Å (middle row), and >3 Å (bottom row). Despite RMSD values exceeding the conventional 2 Å threshold, predicted poses in the 2–3 Å range still maintain similar orientations to the native structures, especially in the core binding motifs.

Table S3. Comparison of the success rates in predicting native poses among three schemes. The comparison was based on the fractions of systems ranked within Top 1, Top 5, and Top 10 across varying distance thresholds used for clustering in the Pose ΔG method.

Method	Threshold (Å)	Top 10	Top 5	Top 1
FULL CRYSTAL	0.5	0.73 ±0.04	0.59 ±0.04	0.40 ±0.04
	1.0	0.75 ±0.04	0.62 ±0.04	0.40 ±0.04
	1.5	0.77 ±0.04	0.64 ±0.04	0.40 ±0.04
	2.0	0.76 ±0.04	0.66 ±0.04	0.40 ±0.04
	2.5	0.79 ±0.04	0.69 ±0.04	0.40 ±0.04
	3.0	0.80 ±0.04	0.70 ±0.04	0.40 ±0.04
	3.5	0.79 ±0.04	0.71 ±0.04	0.40 ±0.04
	4.0	0.79 ±0.04	0.72 ±0.04	0.40 ±0.04
	4.5	0.79 ±0.04	0.72 ±0.04	0.40 ±0.04
	5.0	0.78 ±0.04	0.71 ±0.04	0.40 ±0.04
RANDOM CRYSTAL	0.5	0.72 ±0.04	0.60 ±0.04	0.33 ±0.04
	1.0	0.74 ±0.04	0.64 ±0.04	0.33 ±0.04
	1.5	0.76 ±0.04	0.62 ±0.04	0.33 ±0.04
	2.0	0.76 ±0.04	0.61 ±0.04	0.33 ±0.04
	2.5	0.76 ±0.04	0.62 ±0.04	0.33 ±0.04
	3.0	0.77 ±0.04	0.63 ±0.04	0.33 ±0.04
	3.5	0.76 ±0.04	0.65 ±0.04	0.33 ±0.04
	4.0	0.77 ±0.04	0.66 ±0.04	0.33 ±0.04
	4.5	0.76 ±0.04	0.68 ±0.04	0.33 ±0.04
	5.0	0.77 ±0.04	0.68 ±0.04	0.33 ±0.04
DOCKING	0.5	0.60 ±0.04	0.52 ±0.04	0.29 ±0.04
	1.0	0.66 ±0.04	0.54 ±0.04	0.29 ±0.04
	1.5	0.67 ±0.04	0.55 ±0.04	0.29 ±0.04
	2.0	0.72 ±0.04	0.54 ±0.04	0.29 ±0.04
	2.5	0.74 ±0.04	0.54 ±0.04	0.29 ±0.04
	3.0	0.74 ±0.04	0.55 ±0.04	0.29 ±0.04
	3.5	0.73 ±0.04	0.56 ±0.04	0.29 ±0.04
	4.0	0.78 ±0.04	0.59 ±0.04	0.29 ±0.04
	4.5	0.78 ±0.04	0.58 ±0.04	0.29 ±0.04
	5.0	0.80 ±0.04	0.61 ±0.04	0.29 ±0.04

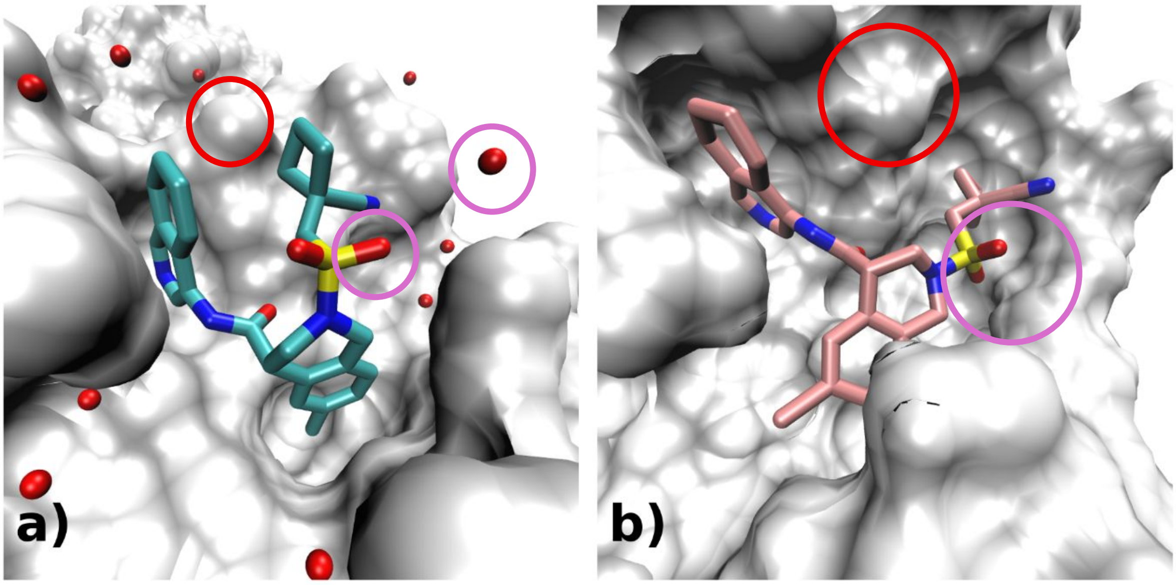

Figure S12. Example of a pose prediction failure for a ligand with a large binding free energy error. This inaccuracy is attributed to the absence of a receptor conformation resembling the crystallographic binding state and to the ligand's conformational flexibility. (a) Crystallographic pose of the ligand; water molecules and the ligand are shown in licorice representation and colored red and cyan, respectively. The cyclobutanyl group is solvent-exposed and unimpeded by neighboring sidechains (red circle), extending toward the bulk water region. In addition, the sulfonyl group appears to form hydrogen-bonding interactions with nearby water molecules (pink circles). (b) Predicted pose of the same ligand, shown in licorice and colored pink. In this pose, a sidechain partially obstructs the region where the cyclobutanyl group would extend (red circle), and both the sulfonyl and cyclobutanyl groups are reoriented inward, facing the protein environment rather than solvent. In both panels, the protein is displayed as a silver molecular surface.

C References

(1) Allen, W. J.; Balius, T. E.; Mukherjee, S.; Brozell, S. R.; Moustakas, D. T.; Lang, P. T.; Case, D. A.; Kuntz, I. D.; Rizzo, R. C. DOCK 6: Impact of New Features and Current Docking Performance. *J. Comput. Chem.* **2015**, *36* (15), 1132–1156. https://doi.org/10.1002/jcc.23905.

(2) Bajusz, D.; Rácz, A.; Héberger, K. Comparison of Data Fusion Methods as Consensus Scores for Ensemble Docking. *Molecules* **2019**, *24* (15), 2690. https://doi.org/10.3390/molecules24152690.

(3) Hart, K. M.; Moeder, K. E.; Ho, C. M. W.; Zimmerman, M. I.; Frederick, T. E.; Bowman, G. R. Designing Small Molecules to Target Cryptic Pockets Yields Both Positive and Negative Allosteric Modulators. *PLoS ONE* **2017**, *12* (6), 1–13. https://doi.org/10.1371/journal.pone.0178678.

(4) Xie, B.; Clark, J. D.; Minh, D. D. L. Efficiency of Stratification for Ensemble Docking Using Reduced Ensembles. *J. Chem. Inf. Model.* **2018**, *58* (9), 1915–1925. https://doi.org/10.1021/acs.jcim.8b00314.
